# Pesticide Exposure of Residents Living Close to Agricultural Fields in the Netherlands: Protocol for an Observational Study

**DOI:** 10.2196/27883

**Published:** 2021-04-28

**Authors:** Daniel M Figueiredo, Esmeralda J M Krop, Jan Duyzer, Rianda M Gerritsen-Ebben, Yvonne M Gooijer, Henk J Holterman, Anke Huss, Cor M J Jacobs, Carla M Kivits, Roel Kruijne, Hans J G J Mol, Arné Oerlemans, Pieter J J Sauer, Paul T J Scheepers, Jan C van de Zande, Erik van den Berg, Marcel Wenneker, Roel C H Vermeulen

**Affiliations:** 1 Institute for Risk Assessment Sciences Utrecht University Utrecht Netherlands; 2 TNO Urban Environment and Safety Utrecht Netherlands; 3 CLM Onderzoek en Advies BV Culemborg Netherlands; 4 Wageningen Plant Research Wageningen University & Research Wageningen Netherlands; 5 Wageningen Environmental Research Wageningen University & Research Wageningen Netherlands; 6 Schuttelaar & Partners Wageningen Netherlands; 7 Wageningen Food Safety Research Wageningen University and Research Wageningen Netherlands; 8 Department for Health Evidence Radboud Institute for Health Sciences Radboud University Medical Center Nijmegen Netherlands; 9 Department of Pediatrics University Medical Center Groningen Groningen Netherlands; 10 Julius Center for Health Sciences and Primary Care University Medical Center Utrecht University Utrecht Netherlands

**Keywords:** pesticides, agriculture, residents, pesticide exposure assessment, environmental samples, biomonitoring, modeling

## Abstract

**Background:**

Application of pesticides in the vicinity of homes has caused concern regarding possible health effects in residents living nearby. However, the high spatiotemporal variation of pesticide levels and lack of knowledge regarding the contribution of exposure routes greatly complicates exposure assessment approaches.

**Objective:**

The objective of this paper was to describe the study protocol of a large exposure survey in the Netherlands assessing pesticide exposure of residents living close (<250 m) to agricultural fields; to better understand possible routes of exposure; to develop an integrative exposure model for residential exposure; and to describe lessons learned.

**Methods:**

We performed an observational study involving residents living in the vicinity of agricultural fields and residents living more than 500 m away from any agricultural fields (control subjects). Residential exposures were measured both during a pesticide use period after a specific application and during the nonuse period for 7 and 2 days, respectively. We collected environmental samples (outdoor and indoor air, dust, and garden and field soils) and personal samples (urine and hand wipes). We also collected data on spraying applications as well as on home characteristics, participants' demographics, and food habits via questionnaires and diaries. Environmental samples were analyzed for 46 prioritized pesticides. Urine samples were analyzed for biomarkers of a subset of 5 pesticides. Alongside the field study, and by taking spray events and environmental data into account, we developed a modeling framework to estimate environmental exposure of residents to pesticides.

**Results:**

Our study was conducted between 2016 and 2019. We assessed 96 homes and 192 participants, including 7 growers and 28 control subjects. We followed 14 pesticide applications, applying 20 active ingredients. We collected 4416 samples: 1018 air, 445 dust (224 vacuumed floor, 221 doormat), 265 soil (238 garden, 27 fields), 2485 urine, 112 hand wipes, and 91 tank mixtures.

**Conclusions:**

To our knowledge, this is the first study on residents’ exposure to pesticides addressing all major nondietary exposure sources and routes (air, soil, dust). Our protocol provides insights on used sampling techniques, the wealth of data collected, developed methods, modeling framework, and lessons learned. Resources and data are open for future collaborations on this important topic.

**International Registered Report Identifier (IRRID):**

RR1-10.2196/27883

## Introduction

### Background

The application of pesticides to agricultural land in the vicinity of homes has raised questions regarding health concerns from residents living nearby. Occupational pesticide exposure has been associated with different health effects including diseases of the respiratory tract [1,2], cancer [3], and neurodegenerative diseases such as Parkinson disease [4,5]. Although residents are likely exposed to lower concentrations than are occupationally exposed individuals, they are continuously exposed because of spray drift and transport of pesticides volatilizing from nearby agricultural land to their homes [6]. In addition, possible accumulation of pesticides in the home environment [7] can contribute to higher and prolonged exposure of those residents [8] compared with urban residents. In addition, in comparison with occupationally exposed workers, more vulnerable groups such as children and the elderly may be exposed in the home environment [9].

While few studies found no clear difference between outdoor air concentrations in urban and rural areas [10], several others have shown that pesticide concentrations in the air are higher close to agricultural fields [11,12] and are higher during the spraying seasons [13,14]. Both results are also true for air and dust in the indoor environment [15,16]. Additionally, when looking at internal dose (measured by biomarkers of exposure), some studies observed significant differences in pesticide exposure levels between urban and rural populations [17-19], while others did not [20,21].

Data on pesticide exposure to residents in the Netherlands are limited, even though approximately 27% of all homes are located within 250 m of at least one cultivated agricultural field as a result of the country's population density and large agricultural sector. Given the variable outcomes in the scientific literature and the lack of information on exposure levels of the Dutch (rural) population due to pesticide use on agricultural fields, the Health Council of the Netherlands advised the government to conduct research in order to fill the above-mentioned gaps of knowledge. For this, the OBO study (“Research on exposure of residents to pesticides”) was conducted.

### Objectives

#### OBO Study

The OBO study aimed to assess the pesticide exposure of residents living close (<250 m) to agricultural fields and to better understand possible routes of environmental exposure. Since most spraying in the Netherlands is done with a downward spraying technique [22-25] and flower bulb cultivation is known to involve a large amount of pesticides [26], the focus was on pesticide exposure among residents living in the vicinity of flower bulb fields. The emphasis of the OBO study was on the assessment of residential pesticide exposure, not on potential adverse health or toxicological effects.

#### This Protocol

To address the above-mentioned objectives, 3 research questions were formulated:

- What are the concentrations of pesticides in the environment of residents living close to agricultural cultivation of flower bulbs compared with those living further away?
- What is the personal exposure to pesticides of residents living close to agricultural cultivation of flower bulbs compared with those living further away?
- What are the sources and routes of exposure contributing to environmental and personal exposure to pesticides in areas of flower bulb cultivation?

In this paper, we describe the OBO study, providing an outline of the methodology used to answer the above-mentioned questions. We provide relevant information, as well as lessons learned, for other researchers planning to set up similar study designs, apply similar methods, and explore collaborations (eg, make use of the collected data in pooled analysis).

### Study Contributions

To the best of our knowledge, this is the first time that a study in the field of residential exposure to pesticides was set up that (1) followed various spraying applications, (2) collected both environmental and personal samples, (3) targeted a wide range of pesticides (ie, insecticides, herbicides, and fungicides), and (4) was performed in different time periods (when pesticides were applied and when pesticides were not applied), allowing the comprehensive study of both spatial and temporal variations in residential pesticide exposure. Contributions of the study to the knowledge base are presented in Textbox 1.

**Contributions of the study to the knowledge base.**

- It produced a FAIR (Findable, Accessible, Interoperable, and Reusable) data set that includes concentrations of many different pesticides in relevant matrices, such as air, dust, soil, and urine. The data set also contains detailed information collected on spraying applications (ie, frequency, mixture applied, quantity, etc). It can be used in future studies for multiple aims, for example to determine the more common pesticide mixtures in the environment or, together with data from other studies, develop robust models to estimate the concentrations of pesticides in certain matrices (eg, indoor home dust as a result of the take-home route).
- It adds to the growing knowledge of pesticide distribution in the environmental matrices and its main determinants, not only for sprayed pesticides but also for some pesticides that were not reported to have been applied.
- It provides valuable and useful information for other researchers and biological monitoring studies regarding toxicokinetics of some pesticides in the human body.
- It provides insights into associations between different matrices (ie, relative pesticide content of air, dust, urine, etc). These results add to the scientific evidence by bringing new knowledge to light.
- A modeling framework was developed that comprises verified models that explain the most relevant pesticide fate processes (eg, spray drift and evaporation), as well as exposure routes (eg, dermal and inhalation). Verification is possible by comparing measured values in the different matrices with modeled values, using all of the collected information on spraying applications and meteorological conditions. This framework or parts of it can be used in future studies as an exposure assessment tool.
- Spray drift and volatilization experiments were performed to increase understanding of the abovementioned processes. These experiments emphasized the importance of drift-reducing nozzles as an exposure reduction factor and the importance of volatilization in pesticide release from the fields.
- It addressed some important current knowledge gaps regarding exposure of residents. Examples are the relative pesticide concentrations in outdoor air and indoor air and which exposure routes contribute most to personal exposure. In our modeling framework, we compared the 4 main exposure routes: contact with surfaces, dust ingestion, dermal contact with the body, and inhalation of gas and particle phase.
- Many of the results can not only be used for policy making in the Netherlands but also be informative for other countries with similar agricultural practices and topographies. Moreover, this protocol—with the description of the study design—can serve as a basis for studies in countries with different agricultural practices but common goals.

## Methods

### Study Design

The OBO study started in January 2016. Enrollment and sample collection were performed in 2016 and 2017. Sample and data analysis were done almost in parallel from mid-2017 to mid-2019. The study focused on flower bulb cultivation and downward spray applications.

**Figure 1:**
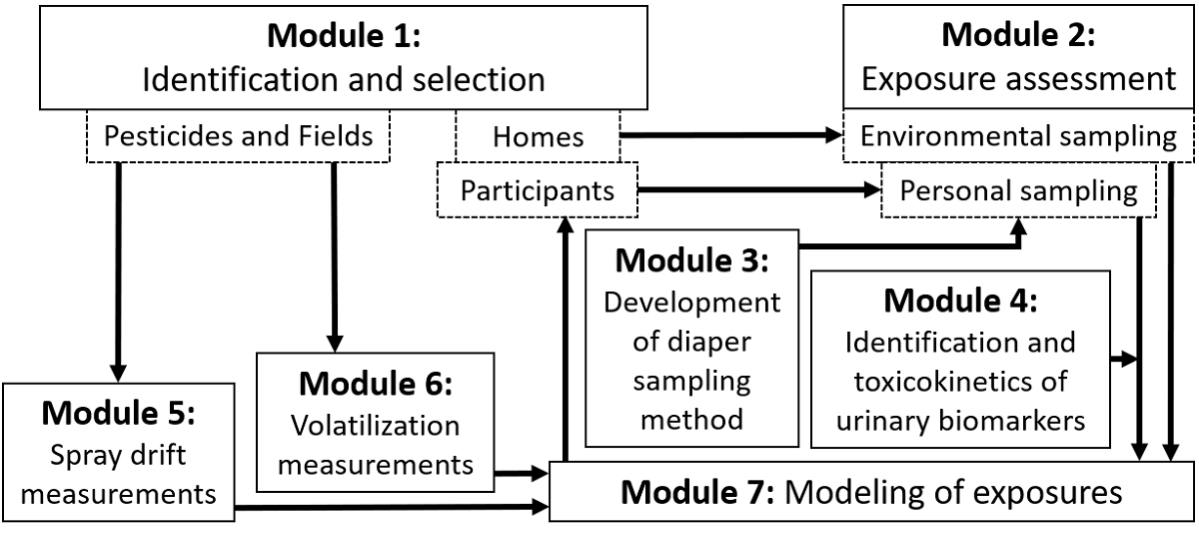
Schematic of the study design.

An exposure assessment strategy was developed to include personal sampling, environmental sampling, and the collection of contextual information. Additional experimental studies were conducted to generate complementary information on methods of urine collection from non–toilet trained infants [27], the toxicokinetics of human metabolites of pesticides [28], as well as experimental applications to better understand pesticide spray drift and volatilization. The study design is shown in Figure 1. At the start of the OBO study (Module 1), the focus was on identification and selection of pesticides to be analyzed and fields, homes, and participants to be studied (ie, residents living in selected homes). In Module 2, exposure assessment was conducted in and around the homes after one of the selected pesticides was sprayed on a selected field. Methods for diaper sampling and assessing personal pesticide exposure were developed in Modules 3 and 4, respectively. On some of the fields, spray drift experiments (Module 5) and volatilization experiments (Module 6) were conducted. Finally, results from Modules 2, 4, 5, and 6 provided input for Module 7, the modeling of exposure for each of the homes (from Module 1). Each module is discussed in more detail below.

#### Module 1: Identification and Selection of Pesticides, Fields, Homes, and Participants

##### Pesticides

**Figure 2:**
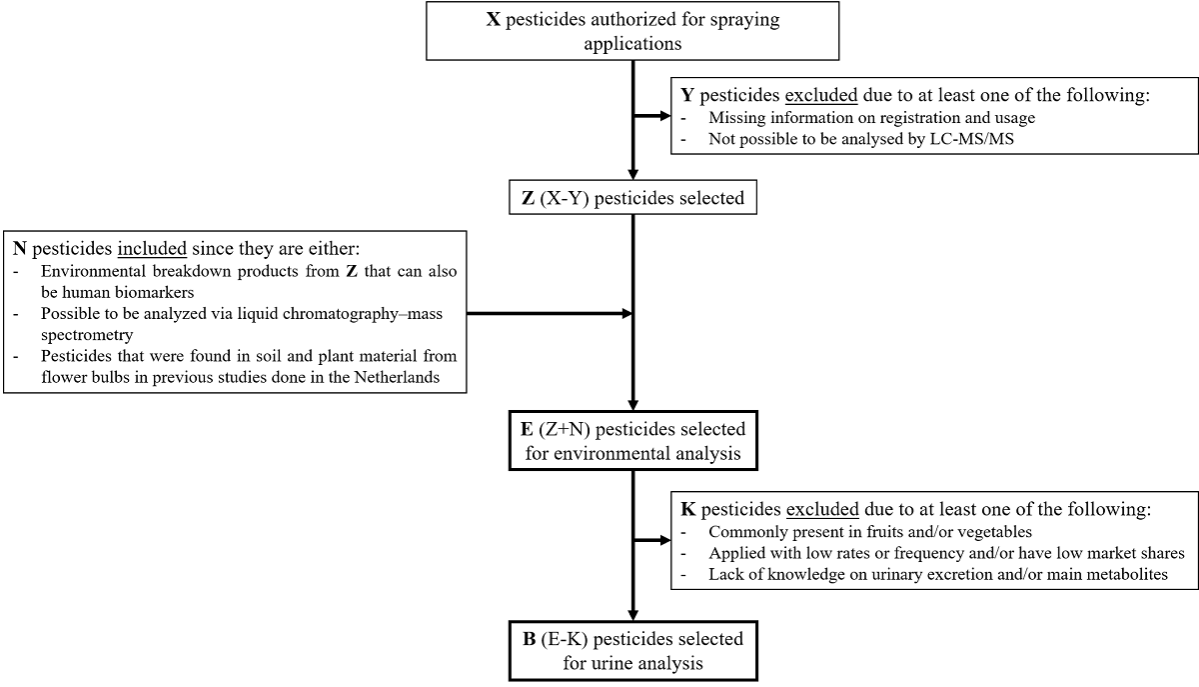
Inclusion and exclusion criteria used in the selection of pesticides to be analyzed in environmental and urine samples. LC-MS/MS: liquid chromatography with tandem mass spectrometry.

In the selection of relevant pesticides to target in our chemical analyses, the main aspects taken into account were (1) information about registration and usage of pesticides on flower bulbs for the year 2015, collected from available data [26] and interviews with growers; (2) existing monitoring data for soil/crops from flower bulb fields; (3) amenability to multiresidue analysis methods; (4) estimated deposition and source strength of emissions from plants and from the top soil layer; (5) estimated dermal exposure and skin absorption potential; and (6) possible exposure originating from other, nonagricultural pesticide use (eg, food consumption) [29]. Detailed inclusion and exclusion criteria are provided in Figure 2.

For the analysis of environmental samples, the aim was to include as many pesticides as possible that are currently applied and/or known to be found in flower bulb fields (see Multimedia Appendix 1), while applying a single analytical method. This was done to reduce costs. For this, we considered multiresidue methods based on liquid chromatography with tandem mass spectrometry (LC-MS/MS) and gas chromatography with tandem mass spectrometry. LC-MS/MS was selected because it covered the largest number of the targeted pesticides. In addition, this method was also more suited to include several relevant degradation products/metabolites. The final selection included 46 prioritized pesticides/metabolites to be measured in air, dust, and soil (see Multimedia Appendix 2).

For the analysis of the urine samples (internal exposure assessment at personal level), the target analyte (biomarker of exposure) in almost all cases was not the parent pesticide but a metabolite formed upon uptake. Of the 46 pesticides selected for environmental measurements, data on their human biomarkers, analytical standards of the potential biomarkers, and methods for their analysis were not yet available. Consequently, as part of the OBO study, data on biomarkers and excretion profiles had to be generated (see Module 4), analytical standards synthesized, and methods for analysis developed. This was a substantial effort and obviously could not be done for all 46 pesticides. For this reason, the assessment of internal exposure was restricted to a subset of 5 pesticides, which should be sufficiently representative to facilitate modeling and extrapolation to other pesticides. Ideally, the pesticides selected for biomonitoring represented the 3 main product types (herbicide, insecticide, and fungicide), different physicochemical properties of the pesticide, and actual spray applications on flower bulbs. We made a short list of 8 prioritized pesticides that were frequently sprayed in bulb fields and could serve as representatives for the whole set and offered good prospects regarding the biomarker analytical challenges. In short, these criteria pertained to factors such as representing different physicochemical properties, pesticide market shares, frequency of application, dosage, vapor pressure, half-life in the environment, and dermal absorption rate. To minimize the influence of dietary contributions on the biomarker levels, we also considered the likelihood of being present in food items.

The 8 selected pesticides were chlorpropham, asulam, flonicamid, acetamiprid, thiacloprid, prochloraz, tebuconazole, and trifloxystrobin. All of these substances were expected to be routinely used by the growers, and most of them, with the exception for chlorpropham, have a low likelihood of dietary exposure compared with other pesticides (see Multimedia Appendix 3). However, as indicated above, because of feasibility constraints, only a maximum of 5 pesticide biomarkers could be analyzed in urine (ie, B in Figure 2 must be equal to 5). Finally, the pesticides (biomarkers) that were selected for biomonitoring were asulam (asulam), carbendazim (methyl 5-hydroxy-2-benzimidazole carbamate [5-HBC]), chlorpropham (4-hydroxychlorpropham-O-sulfonic acid [4-HSA]), prochloraz (2,4,6-trichlorophenol [2,4,6-TCP], and tebuconazole (tebuconazole-1-hydroxy [TEB-OH]).

##### Fields

Selected fields needed to meet the following criteria: (1) residents’ homes were located in the vicinity (within 250 m) of flower bulb fields; (2) growers had a previously defined cultivation plan; and (3) growers were willing to participate and share their spray plan (including product formulation, amount applied, type of nozzle used, and spraying date and hour) with the research team.

Here, we defined location as a place consisting of one or more agricultural fields, with at least one bulb cultivation and surrounded by homes at different distances from those fields. An evaluation comprising a visit to the locations and a meeting with the growers resulted in the final selection of study locations.

It is important to note that there were other fields, besides the selected fields, within 250 m of participating homes. To account for this, growers of all fields near a home (<250 m) that could potentially influence indoor and outdoor environmental pesticide concentrations were asked to share their spraying schemes. In the case of no collaboration (40%), spraying schemes were generated based on type of bulb, weather conditions, and standard spray schemes of the crop type reported by local expert agronomists.

##### Homes

**Figure 3:**
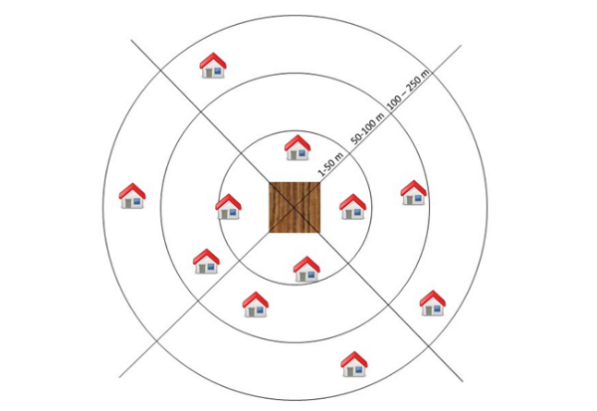
Selection of homes at different distances from selected fields.

Spray applications on a field may expose residents to pesticides through spray drift and volatilization. Homes located within a 50 m distance at the downwind side of the treated field have been described as directly exposed to spray drift [30]. The pesticide deposited on crop and/or soil may volatilize, and this process might affect homes in each direction, especially if they are located within a short distance (ie, up to 250 m) [31]. Therefore, residents living in homes located within 250 m from a selected field were invited to join the study, with, ideally, recruited homes situated at different distances around that field (Figure 3). Control homes were also included in the study. These homes were located in semiurban areas (ie, <1500 residential addresses/km^2^) that were situated within 20 km from a selected field but did not have agricultural fields within a 500 m distance.

##### Participants

Before residents were contacted, the study protocol was approved by the Medical Ethical Committee of the University Medical Center Utrecht (Protocol number NL54727.041.15).

Residents were invited via a letter accompanied by a brochure explaining the study. Interested invitees were interviewed by phone to check if they met the following inclusion criteria: (1) having his/her primary place of residence at the preselected location; (2) having sufficient knowledge of the Dutch language and no cognitive impairment and therefore able to complete the administered questionnaires and communicate with the study assistant; and (3) without a diagnosis of kidney or liver disease, as these could change metabolite formation.

Once enrolled in the study, participants were asked about availability and willingness of further household members (partners and children) to participate.

#### Module 2: Exposure Assessment

For a comprehensive exposure assessment, environmental and urine samples were collected as well as information regarding daily activities, food consumption, building characteristics, and other relevant factors using field forms, questionnaires, and diaries. These data were gathered on two occasions, hereafter referred to as ”measuring campaigns“: during the pesticide use period (UP) and during the nonuse period (NP).

During the UP, environmental samples from homes were collected after a reported spray event on a selected field (Module 1). Outdoor air was sampled for 7 consecutive days because this is the period of time that we expected to see an influence on concentrations due to spray drift (day 1) and evaporation (days 1 to 7) of pesticides. This expectation was based on detailed model calculations of spraying events (using the models described below in Module 7). During the NP, we only expected background concentrations, and therefore we sampled for a shorter period of time (2 days). Biomonitoring was performed on the same days as environmental samples were collected.

For almost all other environmental samples, namely vacuumed floor dust (VFD), dust from a newly placed clean doormat (DDM), windowsill dust, soil from the garden (if one existed), and soil from the selected field, collection took place at the end of the 7-day and 2-day period, respectively, for the UP and the NP. Additionally, in both the UP and the NP, an electrostatic dust collector (EDC) was placed at the start of the measuring campaign and collected at the end.

Regarding personal sampling, morning urine samples were collected daily for 7 consecutive days and hand wipes were taken on the first day of urine collection.

A measurement campaign was set in motion through a system allowing remote initiation of the air pumps once the grower informed the research team that spraying of at least one of the 8 short-listed pesticides was scheduled to begin. This ensured that our sampling periods were aligned with an actual application.

#### Module 3: Development of Diaper Sampling

Self-collection of urine by adults and toilet trained children was done using a 1 L measuring cup and 250 mL plastic jars. To determine the best method for urine collection in non–toilet trained infants (aged 0-3 years), four commonly applied methods were evaluated in a pilot study (in a nonclinical setting). The four methods were (1) free catch, (2) a urine collection pad (Hessels & Grob BV), (3) a urine bag (Urinocol Pediatric, Braun), and (4) a disposable polyacrylate diaper (Pampers Baby Dry size 3, Procter & Gamble). The study examined the success scores of sample collection by parents/caretakers and acceptance scores by infants and parents/caretakers. The most successful and best-accepted method—and also the one that collected a sufficient urine volume (>5 mL) to allow biomarker analyses—was the disposable diaper [27]. This was the method used for urine collection in non–toilet trained infants in this study.

#### Module 4: Identification and Toxicokinetics of Urinary Biomarkers

For most pesticides, metabolism in humans is unknown, and the only available data are derived from animal studies. For urine biomarker analysis, knowledge about the most suitable (specific and sensitive) human biomarker was needed. In addition, in order to link urinary concentrations (internal exposure) to external exposure, knowledge of toxicokinetics and urinary excretion profiles was needed. For this, human volunteer studies were set up for each of the 5 pesticides selected for biomonitoring. Each study involved two independent administrations of the pesticide—one oral and one dermal (2 weeks apart)—to a group comprising 3 males and 3 females. Individual urine samples were collected for 48 hours. First, a biomarker screening was performed for composite urine samples using liquid chromatography–full-scan high-resolution mass spectrometry. For the most suitable biomarker tentatively identified, the analytical standard and its isotopic analog were purchased. In most cases, this required custom synthesis, especially for the isotopic analogs. Following full conformation, dedicated methods for analysis of each biomarker were developed and validated, and all individual samples from each of the volunteers were analyzed. In this way, data on toxicokinetics were generated and conversion factors were derived [28]. The conversion factors were used to estimate pesticide uptake (Module 7) based on measured urinary biomarker concentrations (Module 2).

#### Module 5: Spray Drift Experiments

Spray drift models were developed previously to estimate the environmental fate (ie, spray drift deposition at ground surface and airborne) of pesticides near application areas [32]. However, since residential exposure was not considered during the development of these models, there were knowledge gaps in predicting residential exposure, especially at larger distances (>15 m) from the field and at greater heights (>3 m). To address these gaps, experimental studies were carried out on 6 agricultural fields to study spray drift at longer distances (5 m to 50 m) and greater heights (up to 10 m) as well as the effect of physical barriers. The application techniques for downward spraying were similar to those used in practice. The types of nozzles used were a TeeJet XR11004 (TeeJet Technologies) and agrotop TDXL11004 (agrotop GmbH). These are respectively standard and 90% drift-reducing flat-fan nozzles [33].

For ethical and practical reasons, measurements were performed using a fluorescent tracer instead of pesticides. Experiments were repeated using the aforementioned nozzle types as well as with varying foliage coverage on the field (ie, bare ground to full crop). The results from these studies helped to calibrate the spray drift model, which provided output for use in modeling exposures (Module 7).

#### Module 6: Volatilization Measurements

Pesticide volatilization experiments were also conducted. Two experimental sites were selected based on the defined field and crop types (eg, type of flower bulb) selected in Module 1. In the selected locations, rates of pesticide volatilization from the treated crops and influencing factors were measured on the day of pesticide application and several times during the first week after application. This was achieved by combining measurements of pesticide concentration gradients and on-site meteorological observations, including measurements of turbulence intensity. In addition, the pesticide residues on leaves were determined. Results of these measurements were used to test the volatilization model [34], which provides hourly emissions from fields due to volatilization for use in modeling exposures (Module 7).

#### Module 7: Modeling of Exposures

**Figure 4:**
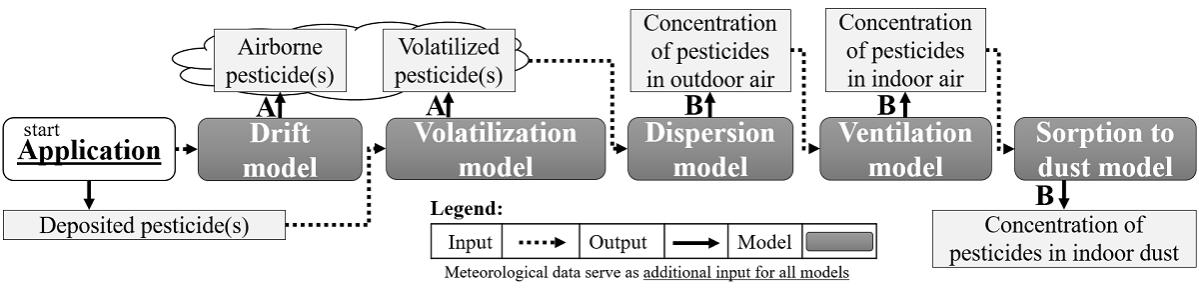
Deterministic modeling framework. (A) Models were calibrated with results from measurements and experimental studies. (B) Modeling steps were verified by comparing predicted and measured concentrations.

In order to select models suitable for assessing the exposure of residents living near fields where pesticides are intensively used, a screening of different models was conducted [35]. The most suitable models were combined into a deterministic modeling framework (Figure 4). Selected models were calibrated with results from measurements and experimental studies (Modules 5 and 6; Figure 4A). Model estimates were verified by comparing predicted concentrations in different media (eg, air, dust, soil) with concentrations measured in and outside homes (Module 2; Figure 4B). Next, the deterministic models were used to estimate pesticide exposure of residents living within 250 m of fields where spraying applications occur (Module 7). In this module, the contributions of different exposure routes to total internal exposure were investigated.

In addition, different factors (eg, personal pesticide use, time spent indoors) that might influence personal exposure were incorporated via statistical modeling techniques.

### Sample Size

To determine our sample size, we performed a power calculation based on National Health and Nutrition Examination Survey data (US Centers for Disease Control and Prevention) on urinary 3-phenoxybenzoic acid (metabolite of pyrethroid pesticides) concentrations. With 200 residents, we estimated that we would reach 80% power at an α=.05 level to detect a 40% to 100% difference between background levels (mean 0.292 µg/L, SD 0.26 µg/L) and exposed individuals, assuming an exposure prevalence of 50% and 100%, respectively. Therefore, we aimed to include 200 residents (roughly 100 homes) in our study.

### Data Collection

#### Measurements

As mentioned in Module 2, different types of environmental and personal samples were collected during this study. All of the different sample and collection procedures are summarized in Table 1. Environmental samples were transported to the laboratory within 48 h after sampling. Air sampling cartridges and dust were stored at 4°C until analysis, while soil and crop samples were stored at –18°C. Analysis of the environmental samples was based on existing methods already available in the consortium laboratories. The methods were slightly adapted to include all 46 pesticides selected and revalidated according to SANTE/11813/2017 (currently SANTE/12682/2019). The latter included establishment of recovery, repeatability, selectivity, and limit of quantification (LOQ; defined as the lowest successfully validated level). For air analysis, the glass fiber filter (trapping airborne particles) and the Amberlite XAD-2 adsorbent (Sigma-Aldrich, Inc; (trapping gas-phase pesticides) were combined and extracted by accelerated solvent extraction using acetonitrile/methanol. After evaporative preconcentration, the pesticides were analyzed by LC-MS/MS. LOQs were 0.01 ng/m^3^ for most pesticides. For the analysis of household dust, soil, and crops, extraction was done by a mixture of water/acetonitrile, followed by a salt-induced phase partitioning technique (QuEChERS) [36,37]. The organic phase was analyzed by LC-MS/MS. LOQs were 1 µg/kg for most pesticides.

Urine samples were transported to the laboratory within 24 h after collection, aliquoted, and then stored at –18°C. For each biomarker, a different dedicated method was developed to obtain optimum performance, which was validated and then applied to sample analysis. In all cases, the isotopically labeled analog of the biomarker was added as an internal standard to the urine aliquot to be analyzed (1 mL to 5 mL) at the start of the sample preparation. For biomarkers of tebuconazole (TEB-OH), prochloraz (2,4,6-TCP), and thiophanate-methyl/carbendazim (5-HBC), an enzymatic deconjugation step was performed. Biomarkers of chlorpropham (4-HSA) and asulam (parent compound) were analyzed directly. For the other biomarkers, extraction/cleanup involved either solid phase extraction or a liquid-liquid partitioning step (QuEChERS-based), followed by an evaporative concentration step. Analysis of the extracts was done by LC-MS/MS under optimized conditions for the respective biomarkers. LOQs were as follows: 0.1 ng/mL for asulam, 4-HSA, and TEB-OH; 0.05 ng/mL for 5-HBC; and 0.25 ng/mL for 2,4,6-TCP.

Not all of the collected samples were analyzed; the unanalyzed samples were kept under appropriate storage conditions for future analysis.

Samples to be analyzed were selected based on the location of the home—to guarantee a good distribution of distances of homes to the selected field—and on the wind direction during the application. This resulted in groups of homes per different distances (ie, homes between 0-50 m, 50-150 m, and 150-250 m) both downwind and upwind of the fields where applications took place. All collected samples from the selected homes were analyzed. For the remaining homes, only DDM was analyzed, providing us with an idea of the distribution of indoor dust concentrations at all locations.

**Table 1:** Samples and collection methods used in the OBO study (”Research on exposure of residents to pesticides”).^a^

Sample	Collection method
	
Outdoor and indoor air	Air was sampled through a standard PM10 inlet and drawn through a glass fiber filter and a tube containing XAD-2 adsorbent (Amberlite XAD-2; Sigma-Aldrich, Inc).
Vacuumed floor dust	A dust sampling sock (Allied Filter Fabrics Pty Ltd) was attached to the hose of a vacuum cleaner to sample for 5 minutes on 4 m^2^ of carpet or 6-8 m^2^ of smooth floor.
Dust from doormat	A clean doormat was deployed for 1 week and then vacuumed on arrival to lab facilities.
Soil from each field and each residential garden	Five uncovered areas of soil were randomly selected and approximately 150 g to 250 g of topsoil were collected per area and combined into a single aggregate soil sample.
Tank mix sample	Duplicate tank mix samples of the spraying liquid were taken directly before and immediately after the spray event in all selected fields (Module 1). Aliquots of the tank samples were stabilized with methanol.
Windowsill dust	Clean wipes were used to collect dust accumulated on windowsill surfaces.
Electrostatic dust collector (EDC)	EDCs were deployed inside each home at the start of the study and collected at the end of the sampling period.
Urine^b^	Spot samples were collected from all participants, except for non–toilet trained toddlers.
Hand wipe^b^	The hand wipe consisted of a facial tissue premoistened with 3 mL of a 50% water/50% ethanol solution.

^a^Analyses were performed using targeted liquid chromatography with tandem mass spectrometry for the main biomarkers of 5 preselected pesticides.

^b^Personal sampling was performed.

#### Questionnaires and Diaries

For each home, the research assistant filled in a field form with building characteristics (see Multimedia Appendix 4), with data on the type of flooring, age of the building and materials used to build it, volume and area, number of floors, type of ventilation system, type of heating, and possible air leakages (eg, cracks). Each participant also completed a questionnaire and diary (see Multimedia Appendix 4) on personal characteristics, socioeconomic position, presence and type of pets, use of medications, education level, type of work/education, whether shoes were worn indoors, and personal use of pesticides. Parents were asked to fill in the questionnaires for their children. Questionnaires were completed before the measurement campaigns started. During measurement campaigns (ie, during both the UP and the NP), participants filled out a daily diary on food intake, hours spent at home and/or elsewhere, and personal use of chemicals, biocides, or pesticides. Via an additional short questionnaire, we checked if items on the original personal questionnaire had changed during the measurement campaigns.

#### Data Management

All data collected from the field study were transferred to the OBO data manager at Utrecht University. Entry of the collected questionnaire data was done using the Castor EDC interface, making our data storage compliant with relevant regulations, such as the General Data Protection Regulation 2016/679, International Organization for Standardization (ISO) 27001, and ISO 9001 [38]. For diaries, we used a tailor-made data entry program. The entry was done in duplicate and then checked against each other (100% check). A third person looked at the differences, and if errors were found, records were rechecked against the original hard copies. Once completed, pseudomized data were used for analyses.

### Ethics: Stakeholder Engagement and Dissemination

The OBO study was commissioned by the Netherlands National Institute for Public Health and the Environment. It was conducted by a consortium of Dutch institutes including Utrecht University, the Netherlands Organization for Applied Research, Wageningen University & Research, Radboud University Medical Center, consulting and communication agency Schuttelaar & Partners, CLM Research and Advice, and Professor PJJ Sauer. The research proposal has been reviewed by a panel of 16 international experts. During the preparation and execution of the OBO study, a stakeholder group advised on all research, communication, and ethical aspects. This group consisted of representatives of public sector policy makers, researchers, the private sector, nongovernmental organizations, and citizens.

## Results

### Identification and Selection

Of the contacted growers of possible selected fields, 17% (12/70) participated in the study. Nine fields were included, encompassing spraying at different crop stages, variability for different meteorological parameters (such as temperature and wind speed), and application of 20 different pesticides (the majority were fungicides [9/20, 45%]). Some fields were sprayed more than once during the UP, which enabled us to follow a total of 14 different primary spraying applications at our selected fields. A total of 80 homes around the selected fields and 16 control homes were included in the study, with a total of 192 participants, of which 39 were younger than 18 years old. An overview is provided in Multimedia Appendix 5.

Initially, the residents of 1778 homes located around the selected fields and 482 control homes were selected and invited to participate. In total, the residents of 80 homes around the selected fields responded and were included in the study (response rate of 4.5%), while the residents of 16 control homes responded and were included (response rate of 3.3%). We were able to have a good spatial distribution of homes around the selected fields: 25% (20/80) of homes were located within 50 m, 43% (34/80) were between 50 m and 150 m, and 33% (26/80) were between 150 m and 250 m from the selected fields. Of the 192 participants, 164 were residents living within 250 m of a selected field. In this group, slightly more than one-half of the participants were female (89/164, 54%) and the average age at participation was 44 years (range 2 to 88 years). Of the 28 participants living in control homes, slightly more than one-half were male (16/28, 57%) and the average age at participation was 50 years (range 12 to 76 years).

We selected 46 active ingredients of pesticides (see Multimedia Appendix 2) for environmental analysis. For biomonitoring, we selected the following 5 pesticide biomarkers: asulam (asulam), carbendazim (5-HBC), chlorpropham (4-HSA), prochloraz (2,4,6-TCP), and tebuconazole (TEB-OH). Carbendazim was not on the initial short list; analyses of indoor dust in the initial phase of the project led to the choice to include this substance in our selection because it was detected in almost all indoor dust samples, often in co-occurrence with thiophanate-methyl. Both are fungicides. Carbendazim, which is no longer registered, arises from the use of thiophanate-methyl, which transforms into carbendazim both in the environment and upon uptake by humans. Thiophanate-methyl has no field spray application in bulb fields but is used for bulb disinfection. It might be emitted from the bulb disinfection site and/or end up in the field upon planting of the bulbs.

### Exposure Assessment

In total, we collected 969 outdoor air samples, 49 indoor air samples, 224 VFD samples, 221 DDM samples, 238 soil samples from residents’ gardens, 27 soil samples from the application fields, 2054 morning urine samples, 431 daytime urine samples, 112 hand wipes, and 91 tank mix samples.

We analyzed approximately one-half of all collected samples. These consisted of 628 outdoor air samples, 43 indoor air samples, 128 VFD samples, 170 DDM samples, 124 soil samples from residents’ gardens, 20 soil samples from the application fields, 791 morning urine samples, and 311 daytime urine samples. All collected hand wipes and tank mix samples were analyzed. The spray events and the respective applied pesticides and tank mixtures are shown in Table 2. The modeling framework was used and verified in all presented locations under several different meteorological conditions (see Multimedia Appendix 5).

**Table 2:** Selected fields and respective applications with reported and measured pesticide concentrations in the tank mixture.

Location, selected field size, measurement campaign, and pesticide sprayed			Grower's self-reported dosage (kg/ha)	Measured dosage (kg/ha)
**A: 2.45 ha**				
	**UP1^a^**			
		Folpet	0.23	nd^b^
		Mancozeb	1.50	nd
		Tebuconazole^c^	0.05	0.06
		Thiacloprid^c^	0.12	0.11
**B: 2.29 ha**				
	**UP1**			
		Flonicamid^c^	0.07	0.06
		Fluopyram^c^	0.08	0.07
		Trifloxystrobin^c^	0.08	0.06
**C: 2.00 ha**				
	**UP1**			
		Chlorpropham^c^	0.80	0.76
		Pendimethalin^c^	0.80	nd
	**UP2^d^**			
		Mancozeb	1.88	nd
		Tebuconazole^c^	0.15	0.15
**D: 1.09 ha**				
	**UP1**			
		Chlorpropham^c^	0.80	0.88
		Pendimethalin^c^	0.80	0.69
	**UP2**			
		Chlorothalonil	0.50	nd
		Esfenvarelate	0.01	nd
		Mancozeb^e^	1.24	nd
		Prochloraz^c,e^	0.16	0.10
**E: 4.58 ha**				
	**UP1**			
		Acetamiprid^c^	0.05	0.07
		Esfenvarelate	0.01	nd
		Mancozeb	1.50	nd
		Mepanipyrim^c^	0.15	0.20
	**UP2**			
		Lambda-cyhalothrin^c^	0.01	nd
		Mancozeb	1.50	nd
		Flonicamid^c^	0.07	0.07
		Tebuconazole^c^	0.08	0.07
**F: 1.83 ha**				
	**UP1**			
		Folpet	0.15	nd
		Tebuconazole^c^	0.15	0.17
	**UP2**			
		Acetamiprid^c^	0.05	0.08
**G: 3.64 ha**				
	**UP1**			
		Asulam^c^	0.20	0.21
		Lambda-cyhalothrin^c^	0.01	0.05
		Metamitron^c^	0.37	0.53
		Mineral oil	4.80	nd
		Quinmerac	0.03	nd
	**UP2**			
		Asulam^c^	0.20	0.21
		Lambda-cyhalothrin^c^	0.01	0.05
		Mancozeb	1.28	nd
		Metamitron^c^	0.37	0.24
		Paraffin oil	4.80	nd
		Pymetrozine^c^	0.10	0.07
		Quinmerac	0.03	nd
**H: 8.40 ha**				
	**UP1**			
		Esfenvarelate	0.01	nd
		Fluopyram^c^	0.08	0.07
		Trifloxystrobin^c^	0.08	0.07
**I: 1.47 ha**				
	**UP1**			
		Trifloxystrobin^c^	0.13	0.09

^a^UP1: pesticide use period 1.

^b^nd: not determined.

^c^Analyzed pesticide.

^d^UP2: pesticide use period 2.

^e^Only applied on part of the field.

## Discussion

There is ongoing concern in the Netherlands regarding the use of pesticides and their potential impact on the environment and human health. In the last decade, several initiatives and regulations have been implemented to reduce the use of pesticides and to reduce the emission of pesticides during (spray drift) and after (volatilization) applications. However, there remains a lack of information on exposure of residents to pesticides coming from agricultural fields. The OBO study was designed to provide comprehensive insight into the exposure of residents and contributing exposure routes.

### Strengths

The design of the OBO study has many strengths. We collected multiple sample types from various matrices in both UPs and NPs. This allowed us (1) to compare exposed locations with control locations in both UPs and NPs; (2) to compare environmental concentrations among exposed locations by UP and distance to fields; (3) to study the interrelationships between concentrations in various matrices (eg, air and dust); (4) to compare biomonitoring results between exposed and control subjects; (5) to relate biomarker levels to environmental concentrations; and (6) to use measurements for model calibration and verification.

Using a single LC-MS/MS method, we managed to determine 46 active substances in the environmental samples. This group of substances covered approximately 60% of the different pesticides registered in the spraying records around the selected fields. The inclusion of 46 different substances allowed us to analyze substances applied in the selected fields, substances applied in other fields in the vicinity, and pesticides with no recorded use in the area, which enabled us to compare patterns between these different use categories.

An emphasis of our study was on modeling of the exposure of residents to pesticides. This resulted in a framework of models that may be useful to also estimate exposure from substances and mixtures that were not included in our study.

### Limitations

One limitation of the study is that participating growers were not blinded (ie, they were informed of the research aim); therefore, one could hypothesize that growers sprayed only under certain conditions (eg, if the wind was blowing away from residential homes or at a very low speed). Of course, in the end, growers will spray when they need to so as to avoid cultivation loss. To account for this, we collected information from multiple applications that occurred not just on the selected field but also on surrounding fields.

Another limitation is that our study focused only on exposure as a result from downward spraying. Information regarding the degree of exposure to pesticides of residents living near crops where sideways or upward spraying techniques are used (such as in fields of fruit trees) is still lacking in the scientific literature and needs to be assessed in future studies. Here, a study design similar to the one used in the OBO study can be used.

Finally, it is important to add that given the low participation rates, the homes and residents included in our study might not be representative of the population living in the vicinity of agricultural fields.

### Lessons From the OBO Study

There are several lessons learned from the OBO study, some related to co-creation (see Textbox 2) and others related to practical aspects (see Textbox 3). We feel that these are relevant to the research community and might help future projects in tackling these a priori.

**Lessons related to co-creation.**

- It was extremely important to have a clear line of communication with the stakeholders and involve them in both the design and the study period. This allowed us to address their concerns upfront. Communication was maintained via presential meetings, and the outcome of the meetings was then transmitted through the entire OBO consortium. We noticed that it was very important to make the aim of the project clear from the start and to check if all stakeholders understood the main goals (ie, managing expectations).
- The collaboration with the branch organizations was also very important as they assisted with the recruitment of growers.
- Information events proved very helpful for dissemination and discussion of results in both the local communities and with the growers.

**Lessons related to practical aspects.**

**Participation and selection**

- Obtaining the participation of selected households (at different distances and in different directions) around selected fields with a diverse population (ie, in age and sex) proved to be a difficult task. Although we achieved a good spatial distribution of homes per distance to selected fields overall, in some locations more than 50% of the homes were located in one of the distance categories (ie, <50 m, 50-150 m, or 150-250 m). Ideally, for each location we would have had one-third of the homes in each of the distance categories. As well, additional requirements, such as multiple persons within a household and the presence of children, made selection of appropriate households challenging.
- The recruitment of growers proved difficult both because of the underlying needs of the project regarding data (eg, a spraying schedule) and because of pressure within the agricultural sector (eg, “is the outcome of the project going to affect me and other growers?”). However, once growers were enrolled in the study, they continued until the end. As mentioned above, the involvement of key branch organizations was very important in this step.
- In the selection of urinary biomarkers, we selected pesticides with a lower exposure background via food intake to make the environmental exposure signal more clear. This was done based on information available in the literature, but it was difficult to achieve high specificity.

**Periodicity of spraying applications**

- Regarding the active air sampling, the logistics were complex, as the exact timing of application was often unknown. Our solution was to install all equipment and wait until the application; however, this created some downtime periods where there were not enough measuring instruments available. Aside from changes in the intended date of application because of changes in meteorological conditions, regular communication with the grower regarding the date of intended application was very useful for the field work planning.

**Analytical standards**

- Synthesis of the analytical standards (and isotope labels) took considerable amount of time and caused a delay in analysis of the urine samples. However, it was difficult to do this in another way. We had to know what was actually sprayed and what was found in the environment; only then could we finalize the selection of the 5 pesticides, start volunteer studies, and, finally, get the biomarkers synthesized. This is important to take into account when setting up a new study.
- In retrospect, the dust samples provided a lot of valuable information regarding presence of various pesticides in the environment. Thus, it would have been useful if we had done a full-scan prescreen of household dust in houses (and fields) of candidate growers and residents' homes. For that, no ethics approval was needed, so we could have done that at a very early stage of the study, during the time when we were working on pesticide selection.

**Exposure of residents to pesticides and communication of results**

- It is important to take into account that we might have only captured a few different exposure scenarios by doing field work. We were constrained by existing meteorological conditions and by the applications that occurred within that time window. As a solution, we used the developed modeling framework to simulate realistic worst-case scenarios by looking into long-term meteorological ranges and different applications settings.
- At the beginning of the project, we promised participants that they would receive feedback on their results, but given the abovementioned time delays, this took a longer time, which resulted in a frustrating process for participants. For future projects, we recommend informing participants a priori of possible delays that might occur.
- Given the very high sensitivity of the methods used, detected exposures may still translate into very low absolute exposures. Therefore, results need to be carefully communicated in order to prevent possible misunderstandings.

### Conclusion

The OBO study can shed light on current and future questions through the materials collected, methods developed, and wealth of data generated. These can, for example, be used for testing model improvements, to put results of other exposure experiments into context, or to develop new hypotheses, thereby also setting the stage for future collaborations.

## Appendix

Multimedia Appendix 1 List of most relevant pesticides in flower bulb cultivation in the Netherlands.

Multimedia Appendix 2 List of 46 pesticides targeted for analysis of environmental samples and their main chemical properties.

Multimedia Appendix 3 Short list of 8 pesticides selected for biomonitoring.

Multimedia Appendix 4 OBO questionnaire, diary, and field form (translated).

Multimedia Appendix 5 Selected fields, measurement campaigns, meteorological conditions, and participating homes and residents.

Multimedia Appendix 6 External peer reviewers report from the National Institute for Public Health and the Environment (RIVM). This report comprises the comments of 16 external reviewers. The comments are in Dutch.

## Abbreviations

2,4,6-TCP: 2,4,6-trichlorophenol
4-HSA: 4-hydroxychlorpropham-O-sulfonic acid
5-HBC: methyl 5-hydroxy-2-benzimidazole carbamate
DDM: dust from a newly placed clean doormat
EDC: electrostatic dust collector
ISO: International Organization for Standardization
LC-MS/MS: liquid chromatography with tandem mass spectrometry
LOQ: limit of quantification
NP: pesticide nonuse period
OBO: Research on exposure of residents to pesticides in the Netherlands
TEB-OH: tebuconazole-1-hydroxy
UP: pesticide use period
VFD: vacuumed floor dust
