# Supplementary material for: Pesticide Exposure of Residents Living Close to Agricultural Fields in the Netherlands: Protocol for an Observational Study
Source: JMIR Res Protoc. 2021 Apr 28;10(4):e27883. doi: 10.2196/27883 (PMC8116989; doi:10.2196/27883)
Supplement: Multimedia Appendix 1 [file resprot_v10i4e27883_app1.docx]

**Supplementary Material 1 -** **List of most relevant pesticides in flower bulb cultivation in The Netherlands**

Table 1. List of most relevant pesticides in flower bulb cultivation in The Netherlands (emphasis on field application in tulip, lily) [situation 2015]

| Pesticides | **Amenability^3^ to** |  |  |
| --- | --- | --- | --- |
| TIER 1. Applied in flower bulbs | **MRM^4^ LC-MS/MS** | **MRM^4^ GC-MS/MS** | **^5^ SRM** |
| acetamiprid | **+** | **-** |  |
| asulam | **+** | **-** |  |
| boscalid | **+** | **+** |  |
| chlorpropham | **+** | **+** |  |
| chlorothalonil^1^ | **-** | **+** |  |
| chloridazon | **+** | **±** |  |
| cyprodinil | **+** | **+** |  |
| deltamethrin | **±** | **+** |  |
| dimethenamide-P | **+** | **+** |  |
| diquat^1^ | **-** | **-** | **+** |
| esfenvaleraat^1^ | **-** | **+** |  |
| flonicamid | **+** | **-** |  |
| fludioxonil | **+** | **+** |  |
| fluopyram | **+** | **+** |  |
| flutolanil^2^ | **+** | **+** |  |
| fosthiazate^2^ | **+** | **±** |  |
| folpet^1^ | **-** | **+** |  |
| glyfosaat^1^ | **-** | **-** | **+** |
| iprodion^1^ | **-** | **+** |  |
| kresoxim - methyl | **+** | **+** |  |
| lambda-cyhalothrin | **±** | **+** |  |
| mancozeb^1^ | **-** | **-** | **+** |
| mepanipyrim | **+** | **+** |  |
| metamitron | **+** | **±** |  |
| metolachlor-S | **+** | **+** |  |
| paraffin oil^1^ | **-** | **-** | **+** |
| pendimethalin | **+** | **+** |  |
| prochloraz | **+** | **±** |  |
| prothioconazole | **+** | **±** |  |
| pymetrozine | **+** | **-** |  |
| spirotetramat | **+** | **+** |  |
| tebuconazole | **+** | **+** |  |
| thiacloprid | **+** | **-** |  |
| tolclofos-methyl^2^ | **+** | **+** |  |
| trifloxystrobin | **+** | **+** |  |
| TIER 2. Data on pesticides found in bulb fields (existing field monitoring data in The Netherlands, bulb fields, 2014, as far as not already mentioned under TIER 1.) | | | |
| azoxystrobin | **+** | **+** |  |
| carbendazim | **+** | **-** |  |
| difenoconazole | **+** | **+** |  |
| dimethomorph | **+** | **+** |  |
| fluopicolide | **+** | **+** |  |
| imidacloprid | **+** | **-** |  |
| linuron | **+** | **+** |  |
| oxamyl | **+** | **-** |  |
| pirimicarb | **+** | **+** |  |
| propamocarb | **+** | **±** |  |
| pyraclostrobin | **+** | **+** |  |
| sulcotrione | **+** | **-** |  |
| terbuthylazine | **+** | **+** |  |
| thiophanate-methyl | **+** | **-** |  |
|  |  |  |  |
| Tier 3. Metabolites from MRM LC-MS/MS amenable pesticides that may also occur as human biomarkers of exposure ^5^ | | | |
| fluopyram-benzamide | **+** | **-** |  |
| desaminometamitron | **+** | **-** |  |
| prothioconazole-desthio | **+** | **-** |  |
| spirotetramat-enol | **+** | **-** |  |
| trifloxystrobin acid (CGA 321113) | **+** | **-** |  |

^1^ not included in environmental measurements

^2^ used for soil treatment

^3^ + means method well suited; ± means moderately suited/not very sensitive; - means not suited/not sensitive

^4^ MRM = multi-residue method (extraction/instrumental method allowing simultaneous measurement of the indicated pesticides)

^5^ SRM = single residue method (dedicated method needed for analysis of that specific pesticide)

^6^ only metabolites for which analytical standards were readily available in 2015 were included for environmental analysis
