# Supplementary material for: Pesticide Exposure of Residents Living Close to Agricultural Fields in the Netherlands: Protocol for an Observational Study
Source: JMIR Res Protoc. 2021 Apr 28;10(4):e27883. doi: 10.2196/27883 (PMC8116989; doi:10.2196/27883)
Supplement: Multimedia Appendix 2 [file resprot_v10i4e27883_app2.docx]

**Supplementary Material 2 - List of targeted pesticides and chemical properties**

Table 2. List of 46 pesticides targeted for analysis of environmental samples

| **Type** | **Pesticides** | **Vap** [mPa] *^a^* | **Log Koa** [-] *^b^* | **dt50 in soil** (days) *^a^* |
| --- | --- | --- | --- | --- |
| **Fungicides** | Azoxystrobin | 1.1E-07 | 1.4E+01 | 1.8E+02 |
|  | Boscalid | 7.2E-04 | 1.3E+01 | 4.8E+02 |
|  | Cyprodinil | 4.9E-01 | 9.5E+00 | 4.5E+01 |
|  | Difenoconazole | 3.3E-05 | 1.4E+01 | 8.5E+01 |
|  | Dimethomorph | 9.9E-01 | 1.1E+01 | 4.4E+01 |
|  | Fludioxonil | 3.9E-04 | 1.2E+01 | 2.1E+01 |
|  | Fluopicolide | 8.0E-04 | 1.5E+01 | 1.4E+02 |
|  | Fluopyram | 4.2E-03 | 1.4E+01 | 1.2E+02 |
|  | Flutolanil | 1.8E+00 | 1.1E+01 | 1.1E+02 |
|  | Kresoxim-methyl | 2.3E-03 | 1.0E+01 | 1.0E+00 |
|  | Mepanipyrim | 2.3E-02 | 9.5E+00 | 5.7E+01 |
|  | Prochloraz | 1.5E-01 | 1.0E+01 | 2.0E+01 |
|  | Propamocarb | 7.3E+03 | 8.3E+00 | 2.0E+01 |
|  | Prothioconazole | 4.5E-09 | 1.4E+01 | 7.7E-01 |
|  | Pyraclostrobin | 2.6E-05 | 1.7E+01 | 3.3E+01 |
|  | Tebuconazole | 1.7E-03 | 1.2E+01 | 4.7E+01 |
|  | Thiophanate-methyl | 9.5E-03 | 8.7E+00 | 2.0E+00 |
|  | Tolclofos-methyl | 5.7E+01 | 6.8E+00 | 7.6E+00 |
|  | Trifloxystrobin | 3.4E-03 | 9.9E+00 | 1.7E+00 |
|  | Fluopyram-benzamide *^(t)^* | 4.2E-03 | 6.8E+00 | 8.6E+00 |
|  | Prothioconazole-desthio *^(t)^* | 1.1E-03 | 1.2E+01 | 4.2E+01 |
|  | Trifloxystrobin-acid *^(t)^* | 5.5E-03 | 9.9E+00 | 7.0E+01 |
|  | Carbendazim *** | 1.0E-04 | 1.1E+01 | 2.2E+01 |
| **Herbicides** | Asulam | 1.9E-01 | 9.9E+00 | 9.0E+00 |
|  | Chloridazon | 6.0E-02 | 9.0E+00 | 3.5E+01 |
|  | Chlorpropham | 2.4E+01 | 8.1E+00 | 1.3E+01 |
|  | Dimethenamid-P | 2.5E+00 | 7.6E+00 | 7.0E+00 |
|  | Linuron | 1.9E-01 | 9.8E+00 | 4.8E+01 |
|  | Metamitron | 8.6E-04 | 1.1E+01 | 1.1E+01 |
|  | Pendimethalin | 1.3E+00 | 9.6E+00 | 1.0E+02 |
|  | Metolachlor-S | 4.2E+00 | 9.3E+00 | 2.1E+01 |
|  | Sulcotrione | 5.0E-03 | 1.1E+01 | 3.6E+00 |
|  | Terbuthylazine | 9.0E-02 | 9.0E+00 | 2.2E+01 |
|  | Metamitron-desamino *^(t)^* | 4.5E-04 | 8.7E+00 | 3.1E+01 |
| **Insecticides** | Acetamiprid | 5.9E+00 | 8.1E+00 | 3.0E+00 |
|  | Cyhalotrin-lambda | 4.5E-04 | 1.1E+01 | 2.7E+01 |
|  | Deltamethrin | 1.2E-05 | 9.9E+00 | 2.1E+01 |
|  | Flonicamid | 9.4E-04 | 1.3E+01 | 3.1E+00 |
|  | Fosthiazate | 5.6E-01 | 9.8E+00 | 1.3E+01 |
|  | Imidacloprid | 2.1E-01 | 1.4E+01 | 1.7E+02 |
|  | Oxamyl | 3.1E+01 | 7.5E+00 | 6.0E+00 |
|  | Pirimicarb | 9.7E-01 | 9.2E+00 | 7.3E+01 |
|  | Pymetrozine | 1.8E-03 | 1.1E+01 | 2.3E+01 |
|  | Spirotetramat | 6.0E-06 | 1.4E+01 | 7.0E-01 |
|  | Thiacloprid | 8.0E-07 | 1.0E+01 | 8.1E+00 |
|  | Spirotetramat-enol *^(t)^* | 6.0E-06 | 1.6E+01 | 1.9E+00 |

*^a^ Vapor pressure and Half-life in soil, from Lewis et al. 2016, ^b^ US EPA. 2020 KOAWIN v1.10 estimate, ^(t)^ transformation product, *besides being a fungicide, carbendazim is also a degradation product of thiophanate-methyl.*
