# Supplementary material for: Pesticide Exposure of Residents Living Close to Agricultural Fields in the Netherlands: Protocol for an Observational Study
Source: JMIR Res Protoc. 2021 Apr 28;10(4):e27883. doi: 10.2196/27883 (PMC8116989; doi:10.2196/27883)
Supplement: Multimedia Appendix 3 [file resprot_v10i4e27883_app3.docx]

**Supplementary Material 3 - Short list of eight pesticides**

To create this short list of pesticides we looked at each group (herbicides, insecticides and fungicides) distinctly to choose the most appropriate ones from each group. Our focus was on pesticides which were authorized for spraying applications. We used the registry of the Ctgb to search for pesticides with an authorized use in one or both flower bulb crops tulip and lily; as at the end of 2015. The selection process is described below for each of these groups.

Herbicides

Indices

Dose rate: Metamitron had the highest recommended dose rate and S-metolachlor the lowest.

Market shares: The highest market shares were reported for chlorpropham and pendimethalin (tulip and lily) and for metamitron (tulip). Also with high shares were asulam and S-metolachlor.

Vapor pressure: Low vapor pressure pesticides were asulam and metamitron. The pesticides pendimethalin, dimethenamide-P, S-metolachlor, and chlorpropham had the highest vapor pressures within the herbicide group.

Dermal absorption rates: Within the above group the highest calculated dermal absorption rates were for chlorpropham, S-metolachlor, and dimethenamide-P.

Exposure to herbicides through food is very unlikely, with the exception being maybe chlorpropham (through potatoes). However, the other selection indices were considered to outweigh a potential issue with background exposure through other routes apart from the environmental route.

Based on these indices for the group of herbicides, **chlorpropham and asulam** were considered the most relevant candidates for inclusion in the biomonitoring. Chlorpropham representing a volatile, low to moderate dose rate pesticide, and asulam representing a non-volatile, high dose rate pesticide.

Insecticides

Indices

Dose rate: Thiacloprid had the highest recommended dose rate and lambda-cyhalothrin the lowest.

Market shares: The market shares of these insecticides were generally lower than the market shares of the investigated herbicides. However, an insecticide with relatively high market share in tulip was spirotetramat.

Vapor pressure: Low vapor pressure pesticides were deltamethrin and spirotetramat. The other pesticides have considerably higher vapor pressures.

Dermal absorption rates: Within the above group the highest calculated dermal absorption rates were for acetamiprid, flonicamid, thiacloprid, and pymetrozine.

All insecticides were applied 2, 3 or 4 times, with the exception of lambda-cyhalothrin which was applied 11 times in tulip and 20 times in lilies.

Based on the above indices for the group of seven insecticides, it was proposed to select either **flonicamid, acetamiprid, or thiacloprid** for biomonitoring in the vicinity of the selected experimental fields used for lily cultivation. The choice depended on the product used by the participating grower.

Fungicides

Indices

Dose rate: Prochloraz had the highest recommended dose rate and Fluopyram the lowest.

Market shares: The market shares of these fungicides were generally lower than the market shares of the herbicides proposed and were comparable with the market shares of the insecticides. The highest market shares were reported for prochloraz, fluopyram, and trifloxystrobin (80%; in lily). The fungicides with the highest market share in tulips were boscalid (55%), and tebuconazole (50%).

Vapor pressure: Low vapor pressure pesticides were prothioconazole and boscalid. The other pesticides have considerably higher vapor pressures.

Dermal absorption rates: Within the above group the highest calculated dermal absorption rates were for tebuconazole, prochloraz, and prothioconazole.

All fungicides were applied 3 to 5 times in tulip, and up to 6 times in lily. The risk of background exposure from food intake was high for boscalid and was also relatively high for tebuconazole, fluopyram, and to a lesser extent for trifloxystrobin and prochloraz.

Based on the above information for the eight identified fungicides, it was proposed to select **prochloraz** for the biomonitoring in the vicinity of the selected experimental fields used for lily cultivation. For tulip, **tebuconazole** was proposed. **Trifloxystrobin** would be a third candidate because it was used both in tulips and lilies. Similar to the insecticides, the choice will depend on the pesticides used by the participating growers.

The final short-list of eight pesticides (active ingredients) is presented in Table 3.

Table 3. Short list of pesticides pre-selected for biomonitoring

| Product use | Active ingredient | Product(s) |
| --- | --- | --- |
| Herbicide | chlorpropham | Intruder, Certis Chloor IPC 40% Vloeibaar |
|  | asulam | Asulox |
| Insecticide | flonicamid | Teppeki |
|  | acetamiprid | Gazelle |
|  | thiacloprid | Calypso |
| Fungicide | prochloraz | Mirage plus 570 SC  Allure vloeibaar |
|  | trifloxystrobin | Luna sensation (also contains fluopyram); Flint |
|  | tebuconazole | Spirit (also contains folpet)  Luna experience (also contains fluopyram) |
