# Supplementary material for: Pesticide Exposure of Residents Living Close to Agricultural Fields in the Netherlands: Protocol for an Observational Study
Source: JMIR Res Protoc. 2021 Apr 28;10(4):e27883. doi: 10.2196/27883 (PMC8116989; doi:10.2196/27883)
Supplement: Multimedia Appendix 4 [file resprot_v10i4e27883_app4.docx]

**Supplementary Material 4 – OBO Questionnaire, diary and field form (*translated*)**

The original documents were written in the dutch language. The material here presented is a translation of the original documents (without formatting).

**Questionnaire**

**About this questionnaire**

This questionnaire includes questions regarding your living situation, lifestyle habits and your work/company and/or study. Also, questions are asked about medicine use, ownership of pets, smoking habits and the use of pesticides. This questionnaire is intended for persons of 16 years and older. The total time needed to complete this questionnaire is 15 to 20 minutes. All your answers will be handled with confidentiality and will be processed and reported in coded form.

In this questionnaire you will encounter different types of questions:

Questions where you can tick one or more boxes or where you can write your answer out on a line, or both. Next, there can be questions where you are asked to enter numbers in boxes.

**Instructions for completing this questionnaire:**

- Please write with a blue or black pen, not with a thick marker.
- Please write all open questions in block letters.
- Note the arrows (=>) which might occur after an answer. An arrow is followed with an explanation.
- If you make a mistake, please check the box with the correct answer and circle the correct box. Do not use correction fluids.
- If you want to make a comment or clarify your answer, you can write next to the question.

If questions are not clear or if you need help with the completion of this questionnaire, you can contact the research assistant through phone or email [contact below].

**PART 1 general questions**

1. What is your date of birth?
   _ _ _ _ _ _ _ _
   day month year
2. What is your gender?
   Male
   Female
3. What is your height?
   _ _ _
   cm
4. What is your weight?
   _ _ _
   kg
5. What is the highest level of education you completed?
   None
   Primary school
   etc

**Questions 6 and 7 only to be filed in by women**

1. Do you currently breast-feed?
   Yes
   No
2. For how long have you been breast-feeding?
   _ _
   months

**PART 2 questions about your living environment and lifestyle habits**

1. Did you have pets in the past 6 months? (excluding poultry/cattle)
   Yes
   No => *please continue with question 10*
2. Which pets did you own in the past 6 months? Please also provide the number of pets per kind of pet. [several answers possible]
   Dog (amount)
   Cat (amount)
   Rodent (amount)
   Bird (amount)
   Other (name and amount)
3. Have you been in direct contact with poultry during the past 6 months?
   Yes
   No
4. Did you use tobacco products and/or e-cigarettes during the past 6 months?
   Yes
   No =>  *please continue with question 13*
5. Which tobacco products and/or e-cigarettes did you use during the past 6 months?
   Cigarettes (amount/day)
   Shag (amount/day)
   Cigars (amount/day)
   e-cigarette (amount/day)
   Other (amount/day)
6. Did you eat homegrown vegetables, fruit and/or herbs during the past 6 months?
   Yes
   No => *please continue with question 16*
7. Indicate per season what proportion of homegrown vegetables, fruit and/or herbs you ate during the past 6 months. [check applicable boxes]

|  | (Almost) all | About 3/4 | About half | About 1/4 | (Almost) nothing |
| --- | --- | --- | --- | --- | --- |
| Spring |  |  |  |  |  |
| Summer |  |  |  |  |  |
| Autumn |  |  |  |  |  |
| Winter |  |  |  |  |  |

1. Did you use groundwater for watering your vegetable garden during the past 6 months?
   Yes
   No
2. What happens to your footwear [usually] when you come from outside and you enter the house?
   I keep my footwear on inside
   I leave my footwear in the hallway/kitchen/garage/shed
   I leave my footwear outside
   Other, namely:
3. What happens with the footwear of other persons living in your household when they come from outside and they enter the house?
   They keep their footwear on inside
   They leave their footwear in the hallway/kitchen/garage/shed
   They leave their footwear outside
   Other, namely:
4. Where do you usually dry the laundry during Summer?
   Inside
   Outside
5. Please indicate on average per day how many hours you did dry your laundry outside during the past 6 months?
   0-1 hours a day
   1-2 hours a day
   2-4 hours a day
   4-8 hours a day
   8 hours or more/day
6. How often have you smelled pesticides in or around your home during the past 6 months?
   Never => *please continue with question 22*
   Less than once a month
   1 to 2 times a month
   2 to 3 times a month
   More than 4 times a month
7. To what extend were you bothered by the smell of pesticides in or around your home during the past 6 months? [please provide a number on a scale from 1 to 10, 1= not bothered, 10=very much bothered]
   _ _

**PART 3 questions regarding medicine use**

1. Did you use medicine(s) on doctor’s prescription during the past 6 months?
   Yes
   No => *please continue with question 24*I do not want to answer this question => *please continue with question 24*
2. Which medicine(s), only on doctor’s prescription, did you use during the past 6 months? (if you do not remember the name of the medicine please indicate what you used the medicine for)
   ______________________
3. Did you use medicine(s) and/or homeopathic substances without prescription during the past 6 months? (e.g. paracetamol, nicotine patches)
   Yes
   No => *please continue with question 26*I do not want to answer this question => *please continue with question 26*
4. Which medicine(s), without prescription, did you use during the past 6 months? (if you do not remember the name of the medicine please indicate what you used the medicine for)
   ______________________

**PART 4 questions about the house**

1. What is the year of construction of the house you are currently living in? (give an estimation if you do not know the exact year)
   _ _ _ _
    year
2. How long do you live in the house you are currently living in? (1 if you live here less than 12 months)
    _ _
    years
3. Please indicate per room and per season if windows were opened usually during the day.

|  | Spring | Summer | Autumn | Winter |
| --- | --- | --- | --- | --- |
| Living room |  |  |  |  |
| Bathroom |  |  |  |  |
| Kitchen |  |  |  |  |
| Your bedroom |  |  |  |  |
| Other rooms |  |  |  |  |

1. How many people are living in your household without counting yourself?
   Amount of people without counting yourself
   _ _
   Year of birth person 1 _ _ _ _
   Year of birth person 2 _ _ _ _
   etc

**PART 5 questions regarding work, business and/or study**

1. Which of the following situations are applicable to you regarding work and study during the past 6 months? (several answers possible)
   Work, 36 hours per week or more, including own business (farm) and self-employed
   Work, 35 hours per week or less, including a side job or helping on the farm (or other business)
   Fulltime student (high school, university etc)
   Part-time student (e.g. evening education)
   Volunteer work => *please continue with question 49*
   Retired => *please continue with question 49*
   None of the above => *please continue with question 49*
2. Please provide the following information regarding your work and/or education of the past 6 months.
   Company/education 1
   Profession/job description
   Activities/tasks
   Amount of days/week Amount of hours/week
   Zip-code, house number
   Company/education 2
   etc
3. Which modes of transportation do you [usually] use from and to your work/business and/or education? Please indicate the average amount of time you spend during a weekday
   Walking (minutes/day)
   Bicycling (minutes/day)
   Scooter/Motor/Moped (minutes/day)
   Car/Van (minutes/day)
   Tractor/Agricultural vehicle (minutes/day)
   Public transport (minutes/day)
   Other, namely: (minutes/day)
4. On an average weekday do you usually travel between different locations?
   Yes
   No => *please continue with question 35*
5. Through which modes of transportation do you [usually] use during a week/workday? Please indicate the average amount of time you spend during a week/workday.
   Walking (minutes/day)
   Bicycling (minutes/day)
   Scooter/Motor/Moped (minutes/day)
   Car/Van (minutes/day)
   Tractor/Agricultural vehicle (minutes/day)
   Public transport (minutes/day)
   Other, namely: (minutes/day)
6. What is the total area of your business or the business where you are employed? Inclusive all agricultural plots/fields/greenhouses.
   _ _ _
   hectare
   I’m not working at an agricultural business => *please continue with question 39*
7. Please provide the agricultural products of your business or the business where you are employed. Please also indicate from when to when the crops were grown, and whether on open ground or in a greenhouse.

| Agricultural product | From | Till | Open ground | Greenhouse |
| --- | --- | --- | --- | --- |
| *Example: potatoes* | *February 2018* | *April 2018* | *Yes* | *No* |
|  |  |  |  |  |
|  |  |  |  |  |

1. Are there poultry and/or cattle present at your business or the business where you are employed?
   Yes, namely:
   No
2. After which time do you enter the plot [usually] after it has been sprayed by you or other persons?
   0-12 hours
   12-24 hours
   1-2 days
   2-7 days
   7 days or more
3. Did you use pesticides and/or biocides at your work during the past 6 months?
   Yes
   No => *please continue with question 45*
4. Which pesticides have you been using at your work during the past 6 months?
   (please provide the name of the substance and the registration number which can be found on the back of the packing)

| Name of substance | Registration number |
| --- | --- |
|  |  |
|  |  |
|  |  |

1. Please indicate which protective equipment you used if you have been working with pesticides and/or biocides. Also indicate if the protective equipment is being cleaned, thrown away or reused without cleaning.

| Protective equipment | Cleaned | Thrown away | Reused without cleaning |
| --- | --- | --- | --- |
| (reusable) overall or other protective clothing |  |  |  |
| Boots |  |  |  |
| Gloves |  |  |  |
| Mouth cap |  |  |  |
| Full- or half-face mask |  |  |  |
| Eye protection |  |  |  |
| Other, namely |  |  |  |
| I’m not using any protective products when working with pesticides => *continue with question 43* |  |  |  |

1. Where and when do you take your protective clothing off usually, when used during spraying activities?
   Inside in a barrack/shed and directly after spraying
   Inside in a barrack/shed, not directly after the spraying activities
   Outside directly after the spraying activities
   Outside, not directly after the spraying activities
   I take off my protective clothing inside the house
   Other, namely:
2. Please indicate how often the clothing used during spraying is washed together with other clothing, of you or other members of the household.
   Always
   Often
   Sometimes
   Almost never
   Never
3. How are pesticides and/or biocides being applied during your spraying activity?
   With a hand sprayer and/or back sprayer
   With an engine grease gun
   With a spraying machine without (closed) cabin
   With a spraying machine with closed cabin
   Other, namely:
4. Do others perform spraying activities with pesticides and/or biocides in your direct working environment?
   Yes
   No => *please continue with question 49*
5. Which pesticides have others been using at your working environment?
   (please provide the name of the substance and the registration number which can be found on the back of the packing)

| Name of substance | Registration number |
| --- | --- |
|  |  |
|  |  |
|  |  |

1. Please indicate which protective equipment you used when others are working with pesticides and/or biocides. Also indicate if the protective equipment is being cleaned, thrown away or reused without cleaning.

| Protective equipment | Cleaned | Thrown away | Reused without cleaning |
| --- | --- | --- | --- |
| (reusable) overall or other protective clothing |  |  |  |
| Boots |  |  |  |
| Gloves |  |  |  |
| Mouth cap |  |  |  |
| Full- or half-face mask |  |  |  |
| Eye protection |  |  |  |
| Other, namely |  |  |  |
| I’m not using any protective products when others are working with pesticides |  |  |  |

1. Please give a short description of how you and/or others in your direct working environment are handling and using pesticides and/or biocides. (think about filling systems, spraying methods, spraying machines, frequencies, amount of liters, amount of hectares etc)
   __________________

**PART 6 use of pesticides and/or biocides in and around the house**

1. Please indicate per category if you used these products in and around the house during the past 6 months. (usage of the products during work are not included)

|  | Yes | No |
| --- | --- | --- |
| Products for example fleas and ticks by pets |  |  |
| Products for head lice |  |  |
| Products for insects (lice, ants, caterpillars, mosquitos etc) |  |  |
| Products for weeds or moss |  |  |
| Products for mold (for example paint or kit against mold) |  |  |
| Products for snails |  |  |
| Products for rats and mice |  |  |
| Other, namely |  |  |

***=>*** *if you only said “no” at the last question, please continue with question 53*

1. Which pesticides and/or biocides did you use in and around the house during the past 6 months? (name and registration number, which can be found on the packing. If you do not remember the name, please indicate the purpose of the product)

| Name of substance | Registration number |
| --- | --- |
|  |  |
|  |  |
|  |  |

1. Did you use any protective equipment or clothing during the use of pesticides/biocides in and around the house during the past 6 months? Yes, only if I used the pesticides/biocides
   Yes, only if others used pesticides/biocides in my direct living environment
   Yes, both if I and others were using pesticides/biocides in my direct living environment
   No => *please continue with question 53*
2. Which protective equipment or clothing did you use in and around the house during the past 6 months?
   (reusable) overall or other protective clothing
   Boots
   Gloves
   Mouth cap
   Full- or half-face mask
   Eye protection
   other, namely:
3. If you do have other information which you think might be important for your urine measures, please indicate this below:
   __________

**Thank you very much for the completion of this questionnaire!**

**Diary**

**About this dairy**

This dairy includes questions about your food intake, use of pesticides and locations you visited. Also, you are asked to write some numbers and times. For example the time of last toilet visit. Enclosed to this dairy is the protocol for urine collection.

You are asked to fill in this dairy one day before urine collection.

This questionnaire is intended for persons of 16 years and older. The total time needed to complete this dairy is 15 to 20 minutes. All your answers will be handled with confidentiality and will be processed and reported in coded form.

In this diary you will encounter different types of questions:

Questions where you can tick one or more boxes or where you can write your answer out on a line, or both. Next, there can be questions where you are asked to enter numbers in boxes.

**Instructions for completing this questionnaire:**

- Please write with a blue or black pen, not with a thick marker.
- Please write all open questions in block letters.
- Note the arrows (=>) which might occur after an answer. An arrow is followed with an explanation.
- If you make a mistake, please check the box with the correct answer and circle the correct box. Do not use correction fluids.
- If you want to make a comment or clarify your answer, you can write next to the question.

If questions are not clear or if you need help with the completion of this questionnaire, you can contact the research assistant through phone or email [contact below].

**PART 1 before urine collection**

1. Please indicate per location how much time you spend today. (in hours and minutes, leave the box empty when you did not spend any time at that location)

Inside
Own house (including sleeping) [hours/minutes]

Inside at work/company [hours/minutes]

Inside in a shed, garage etc [hours/minutes]

Inside somewhere else, namely [hours/minutes]

Transport

In a vehicle (car/bus etc) [hours/minutes]

On a tractor or other agricultural vehicle [hours/minutes]

On a bicycle or walking [hours/minutes]

Outside

At own garden or street [hours/minutes]

Outside at work/company [hours/minutes]

At agricultural field/orchard [hours/minutes]

Outside somewhere else, namely [hours/minutes]

1. Have you been in direct (skin)contact with pets, poultry or cattle today?
   Yes
   No
   Not sure
2. Did you enlighten the fireplace inside today?
   Yes
   No
3. Please indicate per category if you possibly have been in contact or close to these products today. (usage of the products during work are not included)

|  | Yes | No |
| --- | --- | --- |
| Products for example fleas and ticks by pets |  |  |
| Products for head lice |  |  |
| Products for insects (lice, ants, caterpillars, mosquitos etc) |  |  |
| Products for weeds or moss |  |  |
| Products for mold (for example paint or kit against mold) |  |  |
| Products for snails |  |  |
| Products for rats and mice |  |  |
| Other, namely |  |  |

1. In which rooms did you open any windows and/or doors today? Please indicate for how long the doors and/or windows were open.

|  | Up to 1 hour | 1 to 2 hours | 2 to 4 hours | 4 to 8 hours | 8 hours or more |
| --- | --- | --- | --- | --- | --- |
| Living room |  |  |  |  |  |
| Your bedroom |  |  |  |  |  |
| Bedroom child(ren) |  |  |  |  |  |
| Kitchen |  |  |  |  |  |
| Toilet |  |  |  |  |  |
| Bathroom |  |  |  |  |  |
| Other, namely |  |  |  |  |  |

1. Please indicate your food intake per time stamp of the day, including the amount per food type. For vegetables and fruit, it is asked if it is home grown or biological.

Vegetables

|  | Time of consumption | Total amount in pieces or gram | Homegrown? Y/N | Biological?  Y/N |
| --- | --- | --- | --- | --- |
| Endive |  |  |  |  |
| Eggplant |  |  |  |  |
| Broccoli |  |  |  |  |
| Celery |  |  |  |  |
| Cauliflower |  |  |  |  |
| Beans (all kinds) |  |  |  |  |
| Champignon |  |  |  |  |

Others: vegetable mix, cucumber, cabbage, corn, bell pepper, leeks, lettuce, spinach, tomato, onion, chicory, carrot

Fruit

|  | Time of consumption | Total amount in pieces or gram | Homegrown? Y/N | Biological?  Y/N |
| --- | --- | --- | --- | --- |
| Strawberries |  |  |  |  |
| Apple |  |  |  |  |
| Banana |  |  |  |  |
| Berries (all kinds) |  |  |  |  |
| Blackberries |  |  |  |  |
| Citrus fruits (lemon, orange etc) |  |  |  |  |
| Grapes |  |  |  |  |

Others: raspberries, cherries, kiwi, nectarine, pear, peach

Other food

|  | Time of consumption | Total amount in pieces or gram |
| --- | --- | --- |
| Potatoes |  |  |
| Bread |  |  |
| Eggs |  |  |
| Other wheat products (e.g. cereals, muesli) |  |  |
| Pasta (all kinds) |  |  |
| Rice (all kinds |  |  |
| Superfoods (chia seeds, quinoa, etc) |  |  |

Drinks

|  | Time of consumption | Total amount of glasses |
| --- | --- | --- |
| Soda |  |  |
| Packed juice |  |  |
| Fresh juice |  |  |
| Coffee |  |  |
| Milk (all kinds) |  |  |
| Tea |  |  |
| Water |  |  |
| Wine and beer |  |  |

1. Did you barbecue today?
   Yes
   No
2. Has there been a barbecue or firepit in use at your garden or direct surroundings today?
   Yes
   No
3. Did you use any medicine today? (including paracetamol, cough drops etc)
   Yes
   No => *please continue with question 11*
   I do not want to answer this question => *please continue with question 11*
4. Which medicine did you use today? (if you can not recall the name please indicate for what you used it)
   ____________________
5. If you do have other information which you think might be important for your urine measures, please indicate this below:
   __________

**This was the dairy for today, please answer the questions on the next page tomorrow morning after the urine collection.**

**[Instruction for urine collection]**

Needed: container, cool box, cup
Tip: place the container on top of the toilet seat to ensure you won’t forget it.

**Urine Collection**

1. What is the date of urine collection?
   _ _ _ _ _ _ _ _
   day month year
2. At which time did you collect urine?
    _ _ _ _
   hours minutes
3. What is the time of your last toilet visit before urine collection?
    _ _ _ _
   hours minutes

**Field form**

Dear participant,

Would you be so kind to complete this form before the first house visit? If there are any uncertainties you can leave the question open and the fieldworker will help you during the house visit. During the house visit we would like to sketch a map of your home and garden. If you do have (construction) map we would be very pleased to get a copy. Thanks in advance!

1. What is the surface of the house?

< 40 m^2^

40 – 80 m^2^

80 – 120 m^2^

120 – 160 m^2^

160 - 200 m^2^

> 200 m^2^

1. What is the surface of the living room?

_____ m^2^

1. What is the volume of the house?

< 100 m^3^

100 – 200 m^3^

200 – 300 m^3^

300 - 400 m^3^

400 - 500 m^3^

> 500 m^3^

1. What is the height of the house (building of the house)?

_____m

1. What is the construction year of the house?

__________

1. What is the type of house?

Detached house

Semidetached hosue

Terraced house

Appartment

Corner house

1. Does the house have a sloping or flat roof?

Sloping

Flat

1. What is the construction material of the house?

Brick

Concrete

Wood

Metal

Plastic

1. How many floors has the building in which the house is located?

_____ floors (including ground floor and attic, excluding basement)

1. On which floor are the living room, kitchen and bedrooms located? (0 = ground floor)

Floor living room ________

Floor kitchen ________

Floor bedroom adult 1 ________

Floor bedroom adult 2 ________

Floor bedroom child 1 ________

Floor bedroom child 2 ________

1. Please specify the kitchen type:

Open kitchen (clear open connection between kitchen and living room)

Closed kitchen

1. How is the house supplied with hot water?

District heating

Central heating (gas)

Central heating (electric/solar panels)

Geyser in the kitchen with no ventilation flap (gas)

Geyser in the kitchen with ventilation flap (gas)

Other, namely_____

1. What is the heating system of the house?

Hot air heating

Radiators/underfloor heating

1. How is the house heated?

District heating

Central heating

Separate heating elements on gas

Separate heating elements on wood

Separate heating elements on oil

Fire place

Electrical heating

Other, namely_____

1. Do you use gas for cooking?

No

Yes

1. Is there a hood above the cooking area?

No

Yes, exhaust air is recirculated into the kitchen after passing an active coal filter

Yes, exhaust air is ventilated outside the house

1. Please specify the ventilation type in the house (several answers possible):

Air conditioning

Mechanistic ventilation for incoming air

Is always on

Is sometimes turned on by resident

Mechanistic ventilation for discharge of air

Is always on

Is sometimes turned on by resident

Mechanistic ventilation for incoming air and discharge of air

Is always on

Is sometimes turned on by resident

Natural ventilation

Ventilation grille (passive)

Small (<1500cm^2^) window that can be opened by resident

Large (>1500cm^2^) window that can be opened by resident

Door to the outside

Other, namely ______

1. Are there visible holes/cracks in the house that might influence ventilation?

No

Yes

1. Is the house airtight (with foam rubber)?

No

Yes

1. What type of flooring is used in the house?

Smooth floor (e.g. vinyl, parquet, laminate flooring, tiles)

Smooth floor with carpet (larger than 1 m^2^)

Carpeting

1. How old is the floor (give an estimation if you are not certain)?

_____ years

1. Are there any pets present in the house?

Cat amount:

Dog amount:

Bird amount:

Rodent amount:

Other, namely ______
