# Supplementary material for: Pesticide Exposure of Residents Living Close to Agricultural Fields in the Netherlands: Protocol for an Observational Study
Source: JMIR Res Protoc. 2021 Apr 28;10(4):e27883. doi: 10.2196/27883 (PMC8116989; doi:10.2196/27883)
Supplement: Multimedia Appendix 5 [file resprot_v10i4e27883_app5.docx]

**Supplementary Material 5 – Selected fields, followed applications, meteorological conditions, participating homes and residents**

Table 5. Performed measurements, meteo conditions in spraying day and total number of participants.

*n.a.: not applicable*
