# Supplementary material for: Pesticide Exposure of Residents Living Close to Agricultural Fields in the Netherlands: Protocol for an Observational Study
Source: JMIR Res Protoc. 2021 Apr 28;10(4):e27883. doi: 10.2196/27883 (PMC8116989; doi:10.2196/27883)
Supplement: Multimedia Appendix 6 [file resprot_v10i4e27883_app6.pdf]

Rijksinstituut voor Volksgezondheid
en Milieu
*Ministerie van Volksgezondheid,
Welzijn en Sport*

verslag

A. van Leeuwenhoeklaan 9
3721 MA Bilthoven
Postbus 1
3720 BA Bilthoven
www.bestrijdingsmiddelen-omwonenden.nl

KvK Utrecht 30276683

Onderzoek Bestrijdingsmiddelen en Omwonenden (OBO)
T 030 - 274 2529
info@bestrijdingsmiddelen-omwonenden.nl

Datum
22 december 2014

Bespreking	**Begeleidingsgroep OBO: onderzoeksvoorstel**
Vergaderdatum en -tijd	2 december 2014, 10.00-16.15 uur
Vergaderplaats	Vergadercentrum Vredenburg, Utrecht
Deelnemers	Erik Lebret (voorzitter), Willem Asman, Jessica Broeders, Wim Claessens, Marcel Derks, Toon Driessen, Henk Fahner, Christ Goossens, Elly den Hond, Michael Houbraken, Irene Kreis, Marja Lamoree, Ree Meertens, Jo Ottenheim, Ad Ragas, Conno de Ruijter, Gerard Swaen, Aaldrik Tiktak, Manon Vaal, Bram Verhave & Jan-Paul Zock
Afwezig	Harrie Hoeben, Henk van der Schee, Peter Boogaard & Pieter Spanoghe
Verslag	Rik Bogers, Mark Montforts & Wil Noordik

1 Presentaties WP’s

Er wordt gestart met een korte presentatie (10 min.) van de trekkers van de werkpakketten (WP’s).

2 Voorstellen, verklaring van belangen & reglement van orde

Iedereen stelt zich voor, geen van de uitgesproken belangen zijn conflicterend.
Bij de bespreking van de WP’s opent de rapporteur de discussie door zijn/haar beoordeling toe te lichten, na een korte discussie vat de voorzitter samen.

3 Samenvatting/aanbevelingen WP1: overall design

- Maak duidelijk dat de aanleiding om dit onderzoek te doen gezondheidsrisico’s zijn.

  We onderschrijven het belang van een duidelijke communicatie omtrent de aanleiding van het onderzoek en zullen dit in de documenten en communicatie van het project duidelijk aangeven.

- Begin met bekende gezondheidseffecten richting bepaalde stoffen.

  Het voorstel gaat over het kwantificeren van de blootstelling aan bestrijdingsmiddelen bij omwonenden. De stofkeuze wordt gemaakt op basis van gebruikskarakteristieken (gebruikshoeveelheid , aantal toepassingen) om de kans op meetbare niveaus te verhogen. Hierbij spelen toxiciteit en bekende gezondheidseffecten geen primaire rol.

- Betere beschrijving biobank – wie beheert, welke regels gelden, informed consent geregeld?

  In het projectplan zal een betere beschrijving voor het beheer van de monsters worden opgenomen.
  De biobank wordt tijdens het project beheerd door het instituut waar de monsters worden opgeslagen (RIKILT). De milieubank (milieumonsters) zal tijdelijk worden opgeslagen bij TNO. Na afloop van het project zullen de overgebleven biologische en milieumonsters worden opgeslagen in een centrale bio- en milieubank. Een informed consent wordt voor elke deelnemer verzameld.

- Startdatum aanhouden maar fasering inbouwen.

  We zijn het met de begeleidingsgroep eens dat het tijdpad ambitieus is en dat het inbouwen van een geleidelijke fasering verstandig is. We hebben daarom het protocol aangepast en beginnen in jaar 1 met meetprotocol C, waarin onder andere meetmethodieken geoptimaliseerd zullen worden en waarna bij slechts 1 teelt en 2 locaties veldmetingen gedaan zullen worden. Dit betekent een reductie van de meetinspanning in jaar 1 van 66%. De rest van de metingen zal in jaar 3 van de meetcampagne worden ingehaald.

- Meer aandacht nodig voor fasering en detaillering (zie Overall samenvatting/conclusies/aanbevelingen).

  Voordat het onderzoek zal aanvangen wordt er een veldprotocol uitgewerkt waarin alle werkzaamheden gedetailleerd beschreven zullen worden. Dit zijn echter te veel details voor in het huidige onderzoeksvoorstel.

- Doordat er nog geen stoffen zijn geselecteerd en geen gevalideerde analysemethoden zijn ontwikkeld, zal tijdverlies optreden. Advies te selecteren op bekende metabolieten.

  Zoals hierboven aangegeven zullen we in het eerste jaar beginnen met slechts 1 teelt en een beperkt aantal locaties. De teelt in jaar 1 zal fruitteelt zijn waar de belangrijkste stoffen captan en pirimicarb zijn. Voor deze stoffen zijn de metabolieten reeds bekend. We verwachten dat door de nieuwe fasering het risico op tijdsverlies geminimaliseerd wordt.

- Er moet meer aandacht zijn voor biomonitoring. Dit verdrinkt nu in het modelleerwerk en de milieumetingen - het voorstel moet ingaan op de statistische analyse van de biomonitoring; in de powerberekening wordt een controlegroep genoemd maar deze komt verder niet terug in het voorstel.

  Biomonitoring: Het voorstel is om intensief in te zetten op biomonitoring, waarbij bij 400 mensen op 16 dagen urine wordt verzameld. Om deze metingen te kunnen duiden is het echter van belang dat er ook omgevingsmetingen plaatsvinden. Biologische metingen zijn namelijk vaak minder gevoelig omdat concentraties metabolieten in urine doorgaans lager zijn dan concentraties actieve stof in milieumonsters, en vanwege variatie in natuurlijke achtergrondniveaus.

Controles: Er bestaan in dit onderzoek diverse controles.

- *Mensen zijn hun eigen controle. We bemeten de deelnemers gedurende het hele seizoen waarbij we zowel tijdens actieve toepassingen zullen meten maar ook in periodes zonder toepassingen.*
- *Mensen op grotere afstand dienen als controle ten opzichte van de mensen die dichter bij agrarische gewassen wonen.*
- *Doordat verschillende teelten worden bemeten zullen mensen dicht bij een gewas als controle dienen voor deelnemers die dichtbij een ander gewas wonen, indien de gewassen met verschillende stoffen worden behandeld.*

- Door de demografie op het platteland wordt verwacht dat het niet haalbaar is om 50% jeugdigen te includeren.

  We zullen ons maximaal inspannen om zoveel mogelijk gezinnen met kinderen te includeren. Na het eerste jaar zullen we de streefgetallen beter kunnen inschatten.

- Een segmentatie in typen omwonenden wordt geadviseerd. Er zijn bijv. ook loonwerkers onder omwonenden, die andere belangen hebben.

  Alle omwonenden die voldoen aan de insluitingscriteria kunnen deelnemen aan het onderzoek. Bij de intake vragen we naar het beroep van de mensen en op die manier weten we of men als agrariër, loonwerker of anderszins werkzaam is. Op dit aspect selecteren we dus niet actief, maar we nemen het wel in het onderzoek mee.

- Bij aanvang van het onderzoek duidelijk de beperkte doelstelling (alleen blootstelling) communiceren.

  Het is duidelijk dat voor de deelnemers meer speelt dan alleen de zorg over een eventuele blootstelling. Het is dus van groot belang om dit bij includering goed te communiceren. Dit punt krijgt dan ook expliciet aandacht binnen het werkpakket communicatie.

- De BG vraagt zich af of extreme situaties voldoende gedekt worden. Er zou ook een indruk verkregen moeten worden wat er illegaal gebeurt.

  Er is in het onderzoek aandacht voor extreme situaties:
  - *Locaties worden geselecteerd door expliciet te kiezen voor locaties die door veel agrarische percelen omringd zijn, met genoeg huishoudens in de directe omgeving.*
  - *In de modellering zullen op basis van langdurige weerreeksen ook ongunstige meteorologische omstandigheden worden gesimuleerd, die zowel tijdens als na de bespuiting tot relatief hoge emissies en concentraties kunnen leiden.*
  - *In de driftexperimenten is een van de spuitmethodes een spuitmethode zonder drift reducerende maatregelen en als zodanig worst case voor de huidige omstandigheden.*

- De BG heeft twijfels of het haalbaar is bij aanvang van het spuitseizoen in 2015 te beginnen. Punt van aandacht is om hele spuitseizoenen mee te nemen.

Zoals eerder aangegeven zullen in het eerste jaar de experimentele studies worden uitgevoerd en op beperkte schaal veldmetingen. Het zwaartepunt van de meetcampagne zal in 2016 liggen.

- Er is gesproken over het bewaren van monsters om eventueel achteraf markers van gezondheidseffecten te analyseren. Bezwaar hierbij is dat het om te kleine aantallen gaat om dit zinvol te doen. Mogelijk kan wel achteraf op verboden middelen worden geanalyseerd.

  Er worden in het onderzoek alleen urinemonsters verzameld voor het meten van de blootstelling. Deze monsters zullen worden bewaard voor mogelijke toekomstige analyses. Bij aanvang van het onderzoek wordt echter geen toestemming gevraagd voor het bepalen van gezondheidsparameters. Dit behoort daarom niet tot de mogelijkheden.

4 Samenvatting/aanbevelingen WP2: selectie van locaties, teelten, stoffen

- Kwaliteitscontrole en –borging op de stofselectie ontbreekt. Standaard databases met stofgegevens zijn niet feilloos. Kijk na de preselectie van stoffen kritisch gebruik makend van o.a. CTGB database.

  De kwaliteitscontrole is ook een onderdeel van het werk in dit werkpakket. Dit aspect is ook meegenomen in de totstandkoming van de afzonderlijke databestanden, zoals het bestand met de gebruiksgegevens 2008 (in NMI 3). Na de preselectie zal voor de geselecteerde locaties ook nog specifiek gekeken worden naar de onderliggende data en beschikbare databases. Tevens zal de informatie in het veld worden geverifieerd. De uitgevoerde kwaliteitscontrole zal beschreven worden in de rapportage over het werk in dit WP.

- In de eerste fase sterker inzetten op modellering. Met modellen de locatiekeuze bepalen: waar en wanneer verwacht je stoffen te kunnen meten, en kun je überhaupt iets meten. Begin met stoffen waarvan je weet dat je deze kunt meten, extrapoleer met modellen naar andere stofgroepen.

  De locatiekeuze wordt vooral bepaald door de ligging van percelen van een betreffende teelt en de nabijheid van woonhuizen van omwonenden. Bij de selectie van de stof wordt rekening gehouden met de stofeigenschappen. Met modellen worden oriënterende berekeningen uitgevoerd om een idee te krijgen van de te verwachten concentraties. Bij de keuze van de stoffen wordt ook rekening gehouden met de beschikbaarheid van analysemethoden, en dan met name bij de mogelijke stoffen voor biomonitoring, omdat daar naar geschikte metabolieten gezocht moet worden.

- Houd rekening met de omstandigheden tijdens de bespuiting, o.a. soort spuitkoppen, rijsnelheid, spuitrichting en gebruik cocktails (beïnvloeden druppelgrootte).

Bij de uitvoering van de metingen worden alle relevante gegevens verzameld, zoals de gebruikte spuitdoppen, de rijsnelheid van de spuit, het gewasstadium, de windsnelheid ter plaatse, etc. Dit is ook van belang voor de interpretatie van de metingen en onderdeel van WP3. De invloed van relevante parameters op de verspreiding van stoffen wordt bepaald in protocol C.

- Weeg zorgen van omwonenden mee in de locatiekeuze. Om draagvlak te vergroten kan gedacht worden aan een mobiele eenheid die komt meten op het moment dat omwonenden een zorgwekkende situatie/bespuiting waarnemen.

  In het voorstel worden objectieve en goed kwantificeerbare criteria gebruikt voor de locatiekeuze. Het aantal meetlocaties blijft beperkt, gezien de omvang van het werk per meetlocatie. Het meewegen van de perceptie van omwonenden bij de selectie van locaties en de momenten van bemonstering zou verstorend werken op een doelmatige en efficiënte aanpak van de metingen.

5 Samenvatting/aanbevelingen WP 3a: milieumeting

- Een aantal zaken is nog onvoldoende uitgewerkt om goed te kunnen beoordelen. Het plan oogt ambitieus maar de voorgestelde activiteiten zijn wel noodzakelijk.
  Blootstellingsscenario's moeten verder worden uitgewerkt. Het onderzoeksvoorstel beschrijft niet hoe de verschillende (aggregerende) manieren van blootstelling van omwonenden wordt gezien.

  Dat klopt, we gaan (impliciet) verder met de bouwtekening van de Gezondheidsraad. Daarnaast is goed geluisterd naar de omwonenden en zijn hun ideeën opgetekend en meegenomen.

- Om blootstellingsroute goed te kunnen definiëren, is het nodig meer dan eens per dag te bemonsteren. Binnen een paar uur kunnen namelijk grote verschillen in weersomstandigheden zijn die o.a. verdamping vanaf het gewas beïnvloeden, waardoor de piekblootstelling niet noodzakelijkerwijs direct na de bespuiting is. Achteraf kunnen de monsters geselecteerd worden voor analyse die de dynamiek binnen een dag goed beschrijven.

  Vooral op de eerste dag zijn inderdaad grote fluctuaties te verwachten. Wij gaan op dit moment na in hoeverre wij het monsternemingsprotocol kunnen optimaliseren zodat we hiermee rekening kunnen houden. Er zitten echter beperkingen aan het aantal monsters. Via de omgevingsmetingen onder protocol B brengen we bronsterktes (drift en vervluchtiging), concentraties en relevante weersomstandigheden gedurende een dag in kaart, vanaf het moment van toepassing.

- Houd rekening met deposities via neerslag.

  De droge depositie en de depositie via neerslag worden meegenomen in de monsters die genomen worden van de bodem (en de vegetatie).

- Houd rekening met de omstandigheden tijdens de bespuiting. Zoek de invloed van bepaalde parameters eventueel uit in protocol C.

Er is al redelijk veel kennis in het consortium over de invloed van bepaalde parameters op de emissie en de verspreiding. In protocol C worden aanvullende experimenten uitgevoerd gericht op het verzamelen van kennis over de relatie tussen de toepassing en de blootstelling. Het is daarmee de bedoeling de parameters die deze blootstelling beinvloeden verder in kaart te brengen.

- Let op afspoeling in de directe omgeving (vijvers, zwembaden).

  Zie de opmerking boven over de verschillende routes van blootstelling waarvan wij er in de deze studie, het advies van de Gezondheidsraad opvolgend, een groot maar beperkt aantal bestuderen.

- Geef meer aandacht aan wat er in de woning gebeurt en aan de relatie tussen concentraties buiten en binnen.

  Aan de relatie tussen concentraties buiten en binnen wordt in protocol B aandacht besteed. Op basis van de tegelijkertijd uitgevoerde metingen van de concentratie buiten en binnen ontstaat een eerste statistisch beeld van deze relaties. Daarnaast zullen zoveel mogelijk parameters worden bepaald dan wel op andere wijze vastgelegd die deze relatie bepalen zoals windsnelheid, turbulentie, ventilatie van de woning enz. Deze spelen in het vaststellen van de modellering aan de hand van modellen een belangrijke rol.

6 Samenvatting/aanbevelingen WP 3b: biomonitoring

- Het voorstel moet verder worden uitgewerkt: wat is de (biologische) redenering om op bepaalde momenten urine te verzamelen, waarom zo vaak monsters nodig, waarom ochtendurine, onduidelijk of in protocol B 24-uurs of spotsamples worden verzameld.

  Voor protocol A is het verzamelen van ochtendurine het meest effectief: de metabolieten verzamelen zich in de nacht over een vrij lange periode terwijl er dan geen vochtopname plaatsvindt. Dit resulteert in een geconcentreerde urine waarin gewasbeschermingsmiddelen over de voorgaande periode gevoelig kunnen worden gemeten. Dit wordt twee keer zeven dagen gedaan om een goed beeld te krijgen van mogelijke opname in het spuitseizoen en die het gevolg is van normale routinematige toepassing van middelen zodat we een min of meer representatief beeld krijgen (i.t.t. protocol B waar we aan de teler vragen een toepassing van tevoren aan te kondigen). Protocol B is er onder andere op gericht om een beeld te krijgen van de opname (geïntegreerd over de tijd, relevante opnameroutes en relevante bronnen) die het gevolg is van één aangekondigde toepassing op <50/100m van een woning. Na inhalatie van een spuitnevel kunnen metabolieten van de meeste gewasbeschermingsmiddelen binnen enkele uren in de urine verschijnen. Voor andere routes zal meer tijd verstrijken voordat middelen worden opgenomen en ook de opname zelf zal langzaam verlopen als die bijvoorbeeld via de huid optreedt (door bijvoorbeeld hand-mond gedrag of door absorptie via de huid). Om de opname van al deze processen in kaart te kunnen brengen zullen we de personen vragen om alle urine op te vangen vanaf het (aangekondigde) begin van de toepassing tot en met de volgende ochtendurine (zie protocol

A). Voor jonge kinderen die overdag een luier dragen zullen we de ouders vragen over een periode van 2-3 uur alle urine op te vangen en ook de luier te bewaren. Dit vragen we alleen aan de deelnemers die gedurende de toepassing en na de toepassing thuis zijn. Omdat het volume en de tijdperiode bekend is kunnen we de uitgescheiden hoeveelheid metaboliet terugrekenen naar de totale hoeveelheid opgenomen gewasbeschermingsmiddel. Hier wordt dus wel dezelfde procedure gevolgd als bij het verzamelen van een 24-uurs urine, maar de periode is korter.

- De logistiek van het veldwerk is zeer complex. Het veldwerk is nu (ook financieel) onderbedeeld en verdient meer aandacht. Er is bijv. kans op verwisselen van monsters binnen één huishouden, urineverzameling is moeilijk bij vrouwen en er is kans op besmetting, externe factoren zoals een buurtbarbecue verhogen kreatininegehaltes.

  In het consortium is besproken een veldwerker aan het team toe te voegen met kennis en verantwoordelijkheid over het proces van urine verzamelen, tijdelijke opslag bij de woning, ophalen en portioneren op een centrale locatie, evenals het extraheren/overbrengen van urine uit de materialen die we bij jonge kinderen gaan gebruiken (plaszakje, absorptiefilter en luier). Factoren die van invloed kunnen zijn op het kreatininegehalte zullen worden opgenomen in het dagboekje.

- Het voorstel moet kwaliteitscontrole bij monstername en analyses vermelden.

  Voor de monsterneming van urine en de kwaliteitsborging zal een protocol worden opgesteld met daarbij behorende instructies. Voor de analyse zal de kwaliteitscontrole worden vastgelegd in analyseprotocollen.

- De vertaling van metabolieten naar blootstelling vraagt aandacht, bijv. door een vergelijking met opname via de voeding.

  Voor de vertaalslag van metabolieten naar blootstelling zullen humane data worden gebruik als die voorhanden zijn. Voor het geval humane data niet voorhanden zijn, worden op kleine schaal vrijwilligers blootgesteld aan een dosis overeenkomstig de 'acceptable daily intake' (ADI) in een laboratorium. Deze dosis zal oraal worden gegeven, zodat de bijdrage uit voeding kan worden gekwantificeerd.

- Achtergrondblootstelling beter uitwerken.

  Van alle deelnemers zal tweemaal buiten de toepassingsperiode een ochtendurine worden gevraagd. Uit recente studies in de VS blijkt dat dit een kosteneffectieve manier is om het niveau van de achtergrondblootstelling te bepalen (méér urine verzamelen heeft nauwelijks meerwaarde). Vanuit de geselecteerde gewasbeschermingsmiddelen hebben we nog meer mogelijkheden naar de achtergrondblootstelling te kijken. Dit wordt hieronder verder toegelicht.

- Suggestie om een geschikte controlegroep te zoeken.

Binnen de onderzoeksgroep zijn de deelnemers elkaars controle en van zichzelf (zie ook antwoord onder WP1). Middelen die in de fruitteelt worden toegepast worden immers niet in de bollenteelt gebruikt en vice versa. Zo hebben we over en weer grote groepen die alleen een achtergrondblootstelling hebben. Daarnaast is voorzien in een blootstellingsgradiënt die verloopt met de afstand (van <50 m tot meer dan 250 m) van de woning tot het dichtstbijzijnde veld waar de gewasbeschermingsmiddelen worden toegepast. Verder verzamelen we van iedere deelnemer monsters tijdens het spuitseizoen en daarbuiten. Hierdoor ontstaat goed inzicht in wat lokaal als de achtergrondblootstelling kan worden gezien. Een externe controlegroep zal niet worden geworven, omdat dit onnodig is en niet kosteneffectief.

7 Samenvatting/aanbevelingen WP4: analyse methoden

- Het plan is zeer ambitieus. Er is een betere fasering nodig waarbij in de eerste fase een aantal zaken nader wordt uitgezocht en ontwikkeld (zie hieronder). Bijvoorbeeld, doordat de stofselectie nog niet bekend is, is het lastig een adequate analysemethode op tijd klaar te hebben, en lastig te bepalen of urine de juiste matrix is.

  Er is inmiddels een fasering in het plan aangebracht (zie Gantt chart). Wat betreft de matrix voor biomonitoring: daar is in een eerdere fase naar gekeken. Alternatieven (faeces, uitademingslucht, haar, speeksel, bloed) zijn allemaal om uiteenlopende redenen verworpen. Biomonitoring zal dus met urine als matrix moeten gebeuren. Indien stoffen vrijwel niet via urine worden uitgescheiden kan dat een reden zijn om deze niet te prioriteren.

- Er is kans op veel niet-detecteerbare monsters. Er moet iets gezegd kunnen worden over de verwachte concentraties, o.a. gebruik makend van gepubliceerde gegevens over concentraties in het milieu en door te modelleren. De verwachte concentraties in urine geven aan wat de detectielimiet van een methode moet zijn.

  Uitgangspunt bij de eisen aan de detectielimieten van metabolieten in urine zijn geschatte concentraties door blootstelling via voeding (=achtergrond), en gevonden gehalten in vergelijkbare studies uit de literatuur. Dit betekent detectiegrenzen in de range 0.1-1 ng/mL. Als onderdeel van de stofkeuze wordt hier in meer detail naar gekeken.

- Een planning in de vorm van een figuur zoals een GANTT-chart dient te worden toegevoegd.

  Deze is inmiddels opgesteld en wordt aan het onderzoeksvoorstel toegevoegd.

- Het moet duidelijk zijn waar de monsters geanalyseerd gaan worden. Uit het voorstel lijkt het dat dit bij het Rikilt gebeurt, maar in de presentatie van WP4 blijkt anders.

Gezien het aantal monsters en het benodigde aantal methoden en meetsystemen zal de analyse bij verschillende laboratoria worden uitgevoerd, rekening houdend met de beschikbare technische infrastructuur, al beschikbare methoden en beschikbare capaciteit. Dit zullen naast RIKILT tevens TNO, RadboudUMC, Alterra en mogelijk ook IRAS zijn.

- Vermijden dat analyses in verschillende labs worden gedaan, zelfs als het verschillende matrices betreft. Variatie tussen labs werkt door in de verdere modellering. In elk geval interlab vergelijkingen opnemen.

  Dit is gezien de diversiteit van de type monsters, het grote aantal monsters, en het tijdspad waarin ontwikkeling, validatie en analyse dient te gebeuren niet mogelijk. Er zal uiteraard aandacht worden besteed aan controle op systematische fouten in analyseresultaten die door verschillende laboratoria worden gegenereerd, o.a. door interlab controle van gebruikte standaarden en referentiematerialen. Bij analyse van dezelfde type monsters/extracten door verschillende laboratoria zullen monsters worden uitgewisseld om vergelijkbaarheid te borgen.

- De beperkte vrijwilligersstudie moet verder worden uitgewerkt.

  Het belang van de vrijwilligersstudie werd in de loop van de ontwikkeling van het onderzoeksvoorstel, en met name ook daarna, steeds duidelijker. De verdere uitwerking wordt momenteel gedaan, in samenwerking met WP3b.

- De financiële onderbouwing van een kwart A4 voor een onderdeel van bijna drie miljoen euro is ontoereikend. De indruk bestaat dat het zeer hoog begroot is. Er staan vreemde kostenposten zoals een afschrijving van apparatuur van ruim 1/2 miljoen over twee jaar.

  De kosten voor chemische analyse zijn inderdaad hoog. Het gaat om een grote diversiteit van matrices, een reeks van bestrijdingsmiddelen, afbraakproducten/metabolieten, (zeer) lage detectiegrenzen. Er dient een reeks aan methoden (deels) ontwikkeld te worden en in alle gevallen is (her)validatie noodzakelijk. Het gaat om zeer grote aantallen monsters die in veel gevallen met meerdere methoden geanalyseerd dienen te worden om tenminste de geprioriteerde stoffen te dekken. Het type analyse is arbeidsintensief, vereist een hoog kennisniveau (HBO/academisch), een uitgebreide laboratoriuminfrastructuur en gebruik van zeer kostbare high-end apparatuur (hoge aanschafprijs, relatief korte afschrijftermijn, hoge onderhoudskosten, consumables). Een andere kostenpost zijn de analytische referentiestandaarden van metabolieten die speciaal voor deze studie gesynthetiseerd moeten worden. Als deze kosten worden omgerekend naar een prijs per monster dan liggen de analysekosten rond de twee- tot driehonderd Euro per monster, wat vergelijkbaar is met andere projecten en laboratoria.

  Fasering, eerst een pilot is nog steeds nodig. Noem het fase 1.

  Zie antwoorden op bulletpoints 1 en 3.

8 Samenvatting/aanbevelingen WP5: modellering

- De criteria om bepaalde modellen te selecteren moeten expliciet worden gemaakt. Het is nu onduidelijk of de modellen die de onderzoekers voor ogen hebben, gebruikt kunnen worden voor blootstelling. Het betreft daarnaast verschillende soorten modellen op verschillende tijdschalen.

  De modellen worden op dit moment geïnventariseerd op basis van de mogelijkheden om relevante processen te simuleren en van de benodigde in- en uitvoer. Dat modellen verschillende tijdschalen beslaan is juist en afhankelijk van de doelstelling van de modellering zal een specifieke keuze voor een model worden gemaakt.

- Motiveren waarom stoffen gekozen worden en hoe gegeneraliseerd gaat worden naar andere stoffen. Idem voor teelten; o.a. de grootte van een perceel kan verschillen tussen teelten waardoor resultaten niet zonder meer gegeneraliseerd kunnen worden naar andere teelten.

  De stofkeuze is gemotiveerd in de uitleg onder WP2. Er zal gegeneraliseerd worden op basis van stof-, toepassing-, en teeltkarakteristieken, in combinatie met modellering.

- Onzekerheden van de modellering moeten (in elk geval in de rapportage) worden beschreven, m.b.v. een sensitiviteitsanalyse of formele (kwalitatieve) onzekerheidsanalyse vooraf en achteraf (voor gehele onderzoek, breder dan WP5).

  Het in kaart brengen van onzekerheden in de modelleringen (inclusief gegevensverzameling) is van groot belang. We zullen hiervoor sensitiviteitsanalyses uitvoeren. Het doen van een kwantitatieve onzekerheidsanalyse ligt buiten de huidige scope van het werk. We zullen echter wel systematisch de onzekerheden (kwalitatief, kwantitatief) van de input parameters vastleggen.

- De modellen in het voorstel zijn vooral fate modellen, en receptor (individual based) modellen ontbreken. Dit zijn o.a. modellen die gedrag en de relatie tussen concentraties binnen en buiten beschrijven. De laatste jaren zijn hier goede modellen voor ontwikkeld in de VS o.b.v. voedselconsumptie en activiteiten. PBPK modellen kunnen ook worden gebruikt.

  De modellen die beschreven zijn omvatten zowel fate modellen als individual based modellen. De combinatie van deze modellen is noodzakelijk om de blootstelling van de bron tot aan de receptor goed te kunnen beschrijven. PBPK modelering, in zoverre kinetische factoren bekend zijn, maakt ook expliciet deel uit van het modelleringswerk.

- Waak ervoor dat als er luchtverontreinigingsmodellen worden gebruikt die lange afstanden beschrijven (over landen heen) omdat hier geen inputgegevens voor bestrijdingsmiddelen voor zijn. Beperk tot lokaal transport.

We zullen ons in dit onderzoek beperken tot lokaal transport op een schaal van honderden meters. Het gaat in het onderzoek om blootstelling van mensen als gevolg van gebruik van gewasbeschermingsmiddelen in hun directe woonomgeving; lange afstand transport valt buiten de scope van dit project.

- Tijdschalen van de modellen zijn niet hetzelfde, modellen zijn niet duidelijk gespecificeerd.

  We zijn ons bewust van de verschillende tijdschalen van de verschillende modellen. De modellen die in het onderzoeksvoorstel beschreven staan zijn slechts een indicatie van de mogelijke modellen (zie bovenstaand antwoord). Op basis van de in het consortium aanwezige, brede, kennis van modellen kan een goede keuze van modellen en een combinatie van modellen worden gemaakt.

- Maak verbinding met WP 3.

  WP5 is expliciet verbonden met WP3. De meetgegevens uit WP3 zullen gebruikt worden in WP5 om de deterministische modellen te valideren en te kalibreren vooral wat betreft de drift naar de lucht in de driftmodellen voor neerwaartse bespuitingen en het driftmodel voor fruitteelt. Verder zullen ze expliciet gebruikt worden in de statistische modellen die gebruik maken van de metingen en contextuele informatie zoals verzameld in WP3.

- Evalueer effect van emissie van stroken – Protocol C.

  De vraag is niet geheel duidelijk. Indien het hier gaat om eventueel verlies (emissie) van de tracer van de meetstroken dan achten wij dit minimaal.

- Kijk naar achtergrondconcentraties.

  Zoals in WP1 aangegeven wordt er op verschillende manieren gekeken naar achtergrondniveaus.

  Er bestaan in dit onderzoek diverse controles.
  - *Mensen zijn hun eigen controle. We bemeten de deelnemers door het hele seizoen waarbij we zowel actieve toepassingen zullen bemeten maar ook periodes waarbij geen toepassingen plaatsvinden.*
  - *Mensen op grotere afstand dienen als controle ten opzichte van de mensen die dichterbij agrarische gewassen wonen.*
  - *Door dat verschillende teelten worden bemeten zullen mensen dichtbij een gewas als controle fungeren voor deelnemers die dichtbij een ander gewas wonen.*

  Tevens zullen we luchtmetingen uitvoeren zowel boven- als benedenwinds waarbij de voorbelasting bepaald kan worden.

- Wees expliciet in keuzes stoffen

  Zie antwoorden bij WP2 en WP4.

9 Samenvatting/aanbevelingen WP6: Communicatie

- De uitwerking is te globaal, waardoor het moeilijk te beoordelen is wat er om welke reden gedaan wordt. WP6 kan in de uitwerking goed gaan, maar het voorstel is geen garantie dat dit het geval zal zijn.

  In het voorstel zal helderder worden beschreven wat voor elke fase van het onderzoek (voorbereiding, veldwerk, analyse en publicatie van de resultaten) de vraagstellingen zijn die in WP6 beantwoord zullen worden. Tevens zal een korte beschrijving van de methoden worden opgenomen. Een uitwerking in meer detail zal in de bijlage worden opgenomen.

- Doelstellingen van de communicatie moeten geprioriteerd en geselecteerd worden. De verschillende doeleinden (verwachtingen managen, begrijpelijke boodschap, wekken van vertrouwen, etc.) moeten niet op één hoop worden gegooid. Verschillende doelgroepen vragen verschillende oplossingen. Vooral het wekken van vertrouwen is belangrijk.

  In het communicatieplan wordt duidelijk onderscheid gemaakt tussen de verschillende doelgroepen omdat inderdaad de doelstellingen verschillen. Het onderzoeksvoorstel wordt aangescherpt waarbij per doelgroep de doelstellingen van de communicatie worden benoemd.

- Besteed aandacht aan (diversiteit aan) zorgen en denkmodellen van de betrokken partijen. Doe dit niet alleen als desk research, maar koppel hier een onderzoekscomponent aan door gegevens te verzamelen en te analyseren, en deze te gebruiken om zo nodig tussentijds bij te sturen.
- WP6 is er vooral op gericht om 'in het veld' eerst informatie over kennis/houding/perceptie t.o.v. bestrijdingsmiddelen en het onderzoek te verzamelen en vervolgens een vinger aan de pols te houden hoe men het onderzoek en de communicatie hierover ervaart. Deze activiteiten zijn ondersteunend voor de communicatie over het onderzoek door het RIVM, en niet bedoeld als wetenschappelijk onderzoek. Dat laatste vereist namelijk een aanzienlijk grotere onderzoeksgroep, en maakt deel uit van het gezondheidsonderzoek. Kijk indien mogelijk of een handelingsperspectief geboden kan worden voor de verschillende betrokken partijen.

  WP6 levert de basisinformatie en aanbevelingen, die RIVM communicatie kan gebruiken voor het ontwikkelen van een effectieve communicatiestrategie naar alle stakeholders (en intern betrokkenen).

- Risicoperceptie meer nadruk geven.

  Risicoperceptie wordt expliciet meegenomen in het project. Echter omdat het hier om een geselecteerde groep deelnemers gaat waarbij actief wordt gemeten is dit geen informatie die iets zegt over de risicoperceptie van 'omwonenden' in zijn algemeenheid. Risicoperceptie is voorzien in het gezondheidsonderzoek waarin op grote schaal (duizenden mensen) gekeken wordt naar risico- en blootstellingsperceptie in relatie tot attributie van gezondheidsklachten.

10 Samenvatting/aanbevelingen WP7: veldwerk

- Planning in detail uitwerken.
- De planning (en budget) lijkt realistisch maar een concrete tijdslijn met mijlpalen en producten moet worden toegevoegd in detail uitwerken.

  De planning wordt in detail uitgewerkt in het veldwerkprotocol (zie WP1). Dit zal een concrete tijdslijn met mijlpalen en producten bevatten. Deze wordt ook voor het gehele onderzoek opgesteld.

- Onduidelijk of rekening is gehouden met reistijd in de begroting.

  In de begroting is rekening gehouden met reistijd (uren) en reiskosten (onder materiaalkosten) voor de begeleiders/contactpersonen van de deelnemers (zowel omwonenden als telers). Reiskosten voor veldwerkers zijn in WP3 opgenomen.

- Onderzoekers moeten vooraf rekening houden met het feit dat ze voor niets ter plekke zullen zijn. Op één dag wordt soms meer keren de planning voor bespuitingen aangepast.

  Voor de luchtmetingen maken we gebruik van op afstand te bedienen meetapparatuur. Als een teler op korte termijn besluit om de bespuiting op een ander tijdstip uit te voeren is dat geen probleem. De teler meldt het tijdstip waarop hij begint en vervolgens kunnen de pompen direct aangezet worden. Omwonenden worden benaderd via telefoon, sms of email. We zullen daarom goed met hen afstemmen op welke manier zij het best bereikbaar zijn. Wij zijn ons ervan bewust dat de driftmetingen in protocol B wel voorbereidingstijd kosten. Het plaatsen van meetmateriaal kost ca. 3 uur. In dit geval kan het voorkomen dat de onderzoekers mogelijk voor niets ter plekke zullen zijn, wanneer planning van een bespuiting wordt aangepast of de weersomstandigheden het uitvoeren van geschikte metingen verhinderen.

- De twee partners in WP6 die zijn gericht op telers of omwonenden moeten optimaal communiceren omdat de aankondiging van bespuitingen door telers gebeurt, en bij bewoners metingen plaatsvinden.

  De partijen in het onderzoeksconsortium zijn zeer ervaren in de organisatie en uitvoering van vergelijkbaar veldwerk en beschikken over zowel technische vaardigheden als empathisch vermogen. Dit geldt voor het werk bij omwonenden, en ook voor de contacten met en begeleiding van telers. We zijn ons ervan bewust dat optimale communicatie binnen het onderzoeksteam cruciaal is om werkzaamheden op het juiste moment te laten plaatsvinden en hebben er vertrouwen in dat ons consortium werkzaamheden goed op elkaar afstemt.

11 Overall samenvatting/conclusies/aanbevelingen:

- De begeleidingsgroep adviseert positief over het onderzoeksvoorstel, onder de voorwaarde dat er een betere voorbereiding is voordat de metingen beginnen. Bij het ontbreken van een goede voorbereiding is de kans op mislukking groot.

Een gedetailleerd veldwerkprotocol zal worden uitgewerkt waarin alle details van het werk gespecificeerd worden. Verder wordt er een andere fasering van het project voorgesteld waarin in het eerste jaar grondiger voorbereidend werk wordt gedaan en maar in beperkte mate veldmetingen zullen worden gedaan.

- De planning moet in meer detail worden uitgewerkt.

  De specifieke planning wordt momenteel uitgewerkt inclusief milestones en deliverables.

- Er wordt een fasering in drieën geadviseerd: een inceptiefase, metingen jaar 1, metingen jaar 2.

  Dit advies is overgenomen.

- De selectie van stoffen dient eerst te worden gedaan, omdat de inrichting van veel onderdelen van het onderzoek afhankelijk is van de stoffenselectie, en stoffenselectie zwaar doorwerkt in de kwaliteit van het onderzoek en de planning.

  Zie eerdere antwoorden onder de verschillende relevante WPs. Inderdaad is stofselectie van zeer groot belang. Door de nieuwe fasering van het project is daarvoor nu meer tijd ingeruimd.

- Er wordt gesuggereerd om eerst te modelleren:
  - *voor de locatiekeuze*
  - *om verwachte concentraties stoffen te bepalen en daarop de noodzakelijke analytische ondergrens te baseren*

  Zie antwoord onder WP2.

- Betere onderbouwing van de kosten is noodzakelijk.

  De kosten zijn in detail begroot.

- Houd rekening met de eisen van de METC.

  Een eerste draft versie van de METC aanvraag is inmiddels klaar en naar verwachting zal de METC aanvraag medio januari worden ingediend. Op basis van de raadgeving van de METC zal het protocol mogelijk nog worden aangepast.

- Go-/no go-moment inbouwen.

  Het is niet mogelijk op een eenduidig go / no go moment in te bouwen omdat het protocol bestaat uit verschillende elementen die allemaal hun eigen go / no go momenten hebben. We zullen voordat een vervolgstap wordt genomen in het onderzoek kritisch bekijken of alle elementen voldoende duidelijk zijn om een technisch succes te kunnen garanderen.

- Onderzoek communicatie: topics prioriteren en methode beter onderbouwen. Onderdeel risicoperceptie toevoegen.

  Risicoperceptie van de deelnemers zal worden meegenomen om zorg te dragen voor optimale participatie en verwachtingsmanagement. Echter, gegeven dat het hier om een geselecteerde groep omwonenden gaat die deelnemen aan een zeer intensieve studie verwachten wij niet dat deze informatie inzicht geeft in de risicoperceptie van omwo-

nenden in zijn algemeenheid. Hieraan wordt in het parallel lopende gezondheidsonderzoek zeer uitgebreid aandacht besteed in een daarvoor specifiek opgezette studie. Het lijkt daarom raadzaam dit niet te herhalen binnen het blootstellingsonderzoek maar zeker wel om de ervaringen vanuit het blootstellingsonderoek mee te nemen in dit perceptieonderzoek.

- Zo snel mogelijk aan de gang gaan.

  Het consortium dankt de begeleidingsgroep voor de feedback op het onderzoeksvoorstel. Bovenstaande adviezen worden alle ter harte genomen en zijn hierboven uitgebreider toegelicht bij het commentaar op de afzonderlijke werkpakketten.

12 Sluiting

Om 16.15 uur sluit de voorzitter de bijeenkomst.
